# Understanding the use of telemedicine across different opioid use disorder treatment models: A scoping review

**DOI:** 10.1177/1357633X231195607

**Published:** 2023-09-04

**Authors:** Joseph Tay Wee Teck, Jenna L Butner, Alex Baldacchino

**Affiliations:** 1 DigitAS Project, Population and Behavioural Science, School of Medicine, 7486 University of St Andrews, St Andrews, UK; 2 Forward Leeds and Humankind Charity, Durham, UK; 3 Department of Medicine, 12228 Yale University School of Medicine, New Haven, CT, USA

**Keywords:** Telemedicine, opioid use disorder, medication for opioid use disorder, COVID-19, inclusion health, digital divide, digital inequality

## Abstract

**Introduction:**

The COVID-19 pandemic has instigated the development of telemedicine-mediated provision of medications for opioid use disorder such as buprenorphine and methadone, referred to as TMOUD in this study. As services start to return to pre-pandemic norms, there is a debate around the role of TMOUD as addition to or replacement of the conventional cascade of care for people with opioid use disorder (PWOUD). This scoping review is designed to characterize existing TMOUD services and provide insights to enable a more nuanced discussion on the role of telemedicine in the care of PWOUD.

**Methods:**

The literature search was conducted in OVID Medline, CINAHL, and PsycINFO, from inception up to and including April 2023, using the Joanna Briggs Institute methodology for scoping reviews. The review considered any study design that detailed sufficient descriptive information on a given TMOUD service. A data extraction form was developed to collect and categorize a range of descriptive characteristics of each discrete TMOUD model identified from the obtained articles.

**Results:**

A total of 45 articles met the inclusion criteria, and from this, 40 discrete TMOUD services were identified. In total, 33 services were US-based, three from Canada, and one each from India, Ireland, the UK, and Norway. Through a detailed analysis of TMOUD service characteristics, four models of care were identified. These were TMOUD to facilitate inclusion health, to facilitate transitions in care, to meet complex healthcare needs, and to maintain opioid use disorder (OUD) service resilience.

**Conclusions:**

Characterizing TMOUD according to its functional benefits to PWOUD and OUD services will help support evidence-based policy and practice. Additionally, particular attention is given to how digital exclusion of PWOUD can be mitigated against.

## Introduction

Opioid use disorder (OUD) is defined as a persistent and problematic pattern of opioid use resulting in negative impacts on the individual's daily life and/or functioning.^1^ OUD affects more than 26.8 million people worldwide and accounts for two-thirds of deaths directly caused by drug use disorders.^2^ The COVID-19 pandemic has significantly impacted people with OUD (PWOUD) for example through social isolation, increasing the likelihood of solitary opioid use, increased mental health challenges and gender-based violence, and reduced access to health and social care.^3–7^ There is evidence that PWOUD in the US, particularly African Americans, were at increased risk of COVID-19 and associated adverse outcomes.^8^ Indeed, the COVID-19 pandemic has significantly magnified existing structural and health inequalities that disproportionately impacted people from Black and ethnic minority groups, geographically deprived and/or remote regions, poorer socioeconomic backgrounds, and other marginalized social groups.^9,10^ PWOUD, through their intersecting experiences of social exclusion, discrimination, stigma, and homelessness, are over-represented among these marginalized groups.^11,12^

There is a parallel narrative that the COVID-19 pandemic has introduced several policy and regulatory and technological changes and innovations that has improved the delivery of and access to medications for OUD (MOUD) such as methadone and buprenorphine.^13–15^ Examples of these changes have included the provision of more take-home doses, longer prescriptions, and home delivery of MOUD, mobile methadone dispensing units,^16^ and the provision of MOUD through telemedicine termed TMOUD in this paper.^14,15,17^As pre-pandemic norms of healthcare delivery return, there are calls to retain, invest in, and expand upon TMOUD to increase the efficiency and cost-effectiveness of OUD services, extend the reach of limited numbers of addiction specialists and qualified prescribers, and reduce overdose deaths and other OUD sequelae.^15,18^ There is a robust and comprehensive international evidence base in support of the effectiveness of MOUD such as buprenorphine and methadone to reduce opioid related all-cause mortality, overdose risk, and blood-borne virus transmission.^19,20^ There is also an acknowledgment that urgent systems-wide strategies are required to remove barriers to MOUD,^21–24^ increasing the appeal of TMOUD as a viable intervention in countries experiencing overdose crises.^25^

Nevertheless, within a resource-constrained context,^26^ and applying the lessons learnt during the COVID-19 pandemic,^3,13,25^ there are compelling reasons to apply a critical lens when examining the role of TMOUD in meeting the care needs of PWOUD. Firstly, PWOUD often have multiple and complex unmet care needs including housing, legal and income support, the treatment of co-morbid physical and mental health conditions, and harm reduction interventions to prevent blood-borne virus transmission.^19^ Indeed, a recent Scotland-based study by Lowrie et al.,^27^ where per capita drug-related deaths are similar to the US, found that people experiencing homelessness repeatedly overdosed despite having access to MOUD. The authors attributed this to the inadequacy of single condition care models for PWOUD common in Scotland and worldwide, which may provide MOUD without also considering multimorbidity, frailty, and social determinants of health.^27^

Similarly, Hedden et al.,^28^ raise concerns over the displacement of care that occurred during the COVID-19 pandemic, when key services to address PWOUD needs became inaccessible. For example, in the US, there was a 43% reduction and 25% cessation of syringe service programs (SSPs) nationally at the onset of the pandemic. Emergency departments, a common point of urgent care for PWOUD^29^ and consistent primary care, known to improve health outcomes for this group,^28,30^ both had to restrict access in response to the pandemic. Eaves et al.,^31^ make the point that improved MOUD provision through telemedicine will not adequately mitigate against the structural drivers of drug use and risks, and the loss of wraparound services such as sterile injection equipment, wound care, blood-borne virus screening, pre-exposure prophylaxis, antiviral treatment, and naloxone offered by many community-based SSPs.^16,32^

The adequacy of telehealth to fully meet PWOUD care needs and through this, reduce high-risk opioid use and associated morbidity and mortality is one of the key unknowns in current TMOUD research.^18^ More specifically, are there models of TMOUD that can improve access to wraparound and holistic person-centered care, and are there aspects of this care that are best delivered virtually or in person? The question of telehealth and access to care is further muddied by increasing evidence that Black and ethnic minorities and displaced people experiencing homelessness disproportionately under-utilized TMOUD, yet experienced opioid overdoses more frequently.^18,33^ An expansion of TMOUD services, which occurs at the expense of in-person care for PWOUD, will likely lead to the further marginalization of people who struggle to engage with technology due to affordability, skills, literacy, or other aspects of digital exclusion.^34^

While digital exclusion may explain some of the observed inequality in uptake of TMOUD, there may be another more concerning explanation. There was evidence that clinicians delivering MOUD through telemedicine excluded prospective patients perceived to carry more liability risk due to greater complexity in their care needs.^33^ This included people not already receiving MOUD, those who were taking multiple substances, had significant co-morbidities and/or were experiencing homelessness.^35,36^ Models of telemedicine that encourages the cherry-picking of patients with lower intensity needs may result in widening already unequal OUD care access to those who most need it, thus exacerbating racial and socioeconomic inequality.^37^

There is therefore an argument for a more nuanced approach to capitalizing on the growth of telemedicine and its use in delivering MOUD to improve care for PWOUD. With this in mind, we conducted this scoping review to answer the following questions:

1. What models of TMOUD are currently described in the peer-reviewed literature?
2. How do available TMOUD models cater for the diverse care needs of PWOUD?
3. What are the knowledge gaps in developing TMOUD models, which equitably improve access to OUD care?

## Methods

This study was conducted according to the Joanna Briggs Institute methodology for scoping reviews.^38^ The review findings are reported in accordance with the Preferred Reporting Items for Systematic Reviews and Meta-Analyses (known as PRISMA) extension for scoping reviews.^39^

### Search strategy and selection criteria

Searches were conducted in three databases, OVID Medline, CINAHL, and PsycINFO, from inception up to and including 6 April 2023. Search terms for OUD, problematic opioid use, and medications for OUD such as methadone and buprenorphine were combined with terms for telemedicine, such as telepsychiatry, telehealth, videoconferencing, and remote consultation. An example of the search strategy and terms are included in the Supplemental material. The search strategy was intended to capture a diverse range of TMOUD services reported in the peer-reviewed literature. Consequently, articles were included if they were peer-reviewed, in English, and described service or systems level implementation, or the adaptation of existing services to deliver TMOUD in any setting and involving any population needing care for OUD. The review considered any study design provided sufficient descriptive information of the TMOUD service was available. Articles without abstracts, not peer-reviewed such as commentaries and/or letters, from the grey literature, or describing the use of technology but not directly connected to the delivery of MOUD were excluded. Zotero, an open-source bibliography management tool (v6.0.23) for Mac was used to manage references and remove duplicates.

### Data extraction and synthesis

The retrieved records were screened by title and abstract using the review-management tool, Rayyan, by one author (JT). Full-text articles of the records passing this initial screen were obtained and reviewed for suitability for inclusion (JT). A second reviewer (AB) reviewed randomly selected 10 articles to independently verify their suitability according to the established inclusion criteria. Discrepancies between the two authors on whether an article should be included were resolved by consulting a third reviewer (JB). The final selection of papers was downloaded into the qualitative analysis platform Atlas.ti for Mac (v22.1.0).

Basic data were captured such as the year of publication, the country where the intervention was carried out, whether it was implemented in response to the COVID-19 pandemic, and the study design used. A more detailed data extraction form based on work by Lagisetty et al.,^40^ and Tang et al.,^41^ was developed to collect and categorize specific descriptive characteristics of each discrete TMOUD model identified. This included the treatment setting, existing in-person treatment model, the target population, threshold for care, whether the service was adapted or designed to be virtual, whether there was a hybrid offer (both in person and virtual), exclusion and inclusion criteria for enrollment, medications offered, and contingencies for care continuity. The data were extracted by one reviewer (JT) into a Microsoft Excel spreadsheet. Where possible, missing descriptive data of each TMOUD service was obtained either by contacting the article's author(s) or by looking at the service website. The findings of this review are reported in a narrative format.

## Results

**Figure 1.**
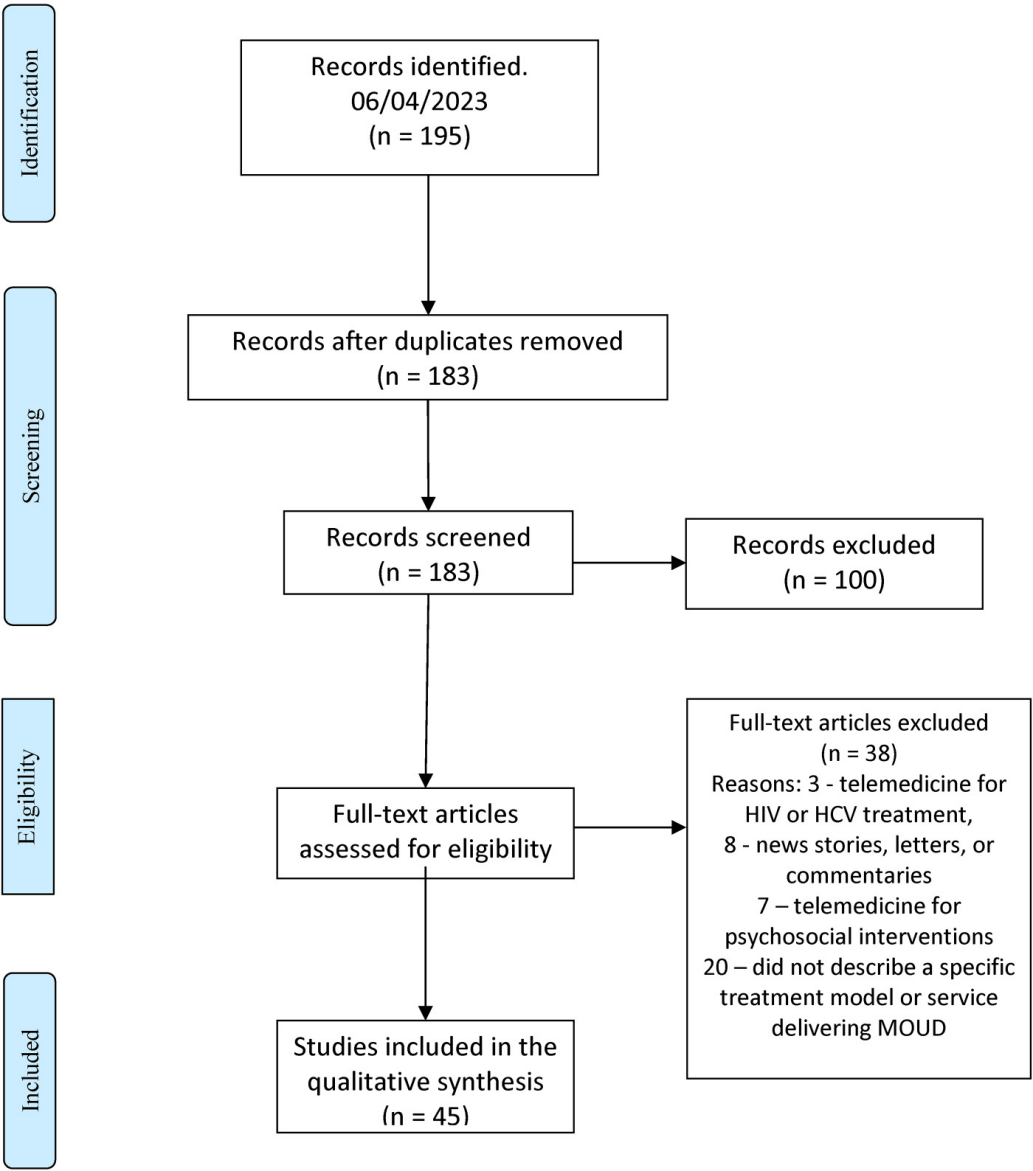
PRISMA diagram.

The search conducted on 6 April 2023, identified 195 records. Deduplication left 183 records screened by title and abstract, of which 100 were excluded due to irrelevance to the topic. A total of 83 full-text articles were then screened, of which 45 met our inclusion criteria (Figure 1).

### Characteristics of the included articles

 Table 1 summarizes the key characteristics of the included papers, and their contribution to this review can be found in the Supplemental Table 1. A total of 36 out of 45 papers (80%) were from the US, and 16 of the included papers described service innovation (16/45; 35%), or service innovation and evaluation (6/45; 13%), making up 49% of the included studies. There were 28 of the 45 papers (62%) that described service innovations in response to the COVID-19 pandemic.

**Table 1.** Characteristics of studies included in the review.

Characteristic	Number of articles (*n* = 45)	Percentage (100%)
Article type		
Case report	2	4.44%
Feasibility study	3	6.67%
National Delphi study	1	2.22%
Non-randomized controlled trial	1	2.22%
Qualitative interviews	3	6.67%
Randomized controlled trial study protocol	2	4.44%
Retrospective data analysis	11	24.44%
Service innovation	16	35.56%
Service innovation and evaluation	6	13.33%
Year of publication		
2017	2	4.44%
2018	2	4.44%
2020	4	8.89%
2021	16	35.56%
2022	13	28.89%
2023	8	17.78%
COVID-19-related service adaptation	28	62.22%
Country of origin		
Canada	3	6.67%
India	1	2.22%
Ireland	2	4.44%
Norway	1	2.22%
UK	2	4.44%
USA	36	80.00%

### Services identified in this review

We identified 40 discrete services described across the 45 included studies. Table 2 summarizes some of the key characteristics of these services, and more detailed information on each service is provided in the Supplemental Tables 2–5. A total of 17 (17/40; 42.5%) services provided care for urban communities, 17 (17/40; 42.5%) for mixed urban-rural settings, and 6 (6/40; 15%) served rural settings exclusively. Most services were from the US (33/40; 82%), and within this, there was a diverse range of service settings from which TMOUD was delivered, including SSPs (8/33; 24%), harm reduction and street-based services (11/33; 33%), emergency departments (7/33; 21%), and hospital-based including perinatal services (9/33; 27%) and primary care (13/33; 39%).

**Table 2.** Characteristics of MOUD services identified in this review.

Service characteristics	Discrete services (*n* = 40)	Percentage
Geographic setting		
Urban	17	42.5
Rural	6	15
Mixed	17	42.5
Threshold of care		
High	4	10
Low	14	35
Unclear/not described	22	55
Linking to community MOUD services	8	20
Target population		
People who inject drugs	7	17.5
People experiencing homelessness	6	15
People with substance use disorder during pregnancy	2	5
People with HIV	4	10
People with HCV	2	5
People involved with criminal justice	3	7.5
People involved with sex work	1	2.5
Service setting(s)		
Syringe service programs	8	20
Harm reduction services	3	7.5
Street-based	8	20
Other community settings	4	10
Emergency departments	7	17.5
Hospital-based (including obstetrics)	9	22.5
Primary care	15	37.5
Dedicated treatment facility	10	25
Prisons	1	2.5
Fully virtual	3	7.5
National or systems-wide	17	42.5
Medication offered		
Buprenorphine s/l	40	100
Methadone solution	8	20
Buprenorphine injectable depot	6	15
Harm reduction interventions		
Take-home naloxone	13	32.5
Overdose awareness and response	13	32.5
Management of polysubstance use (co-occurring SUD such as amphetamine and cocaine) or alcohol	5	12.5
Sterile injection equipment	8	20
BBV testing	6	15
HCV cure	5	12.5
ART-HIV suppression/PrEP	4	10
Wound care	6	15
Fentanyl strips	2	5
Digital overdose response system	1	2.5
Meeting other clinical needs		
Onsite phlebotomy	3	7.5
Flexible medication management protocols such as medication lockers	2	5
Flexible medication management protocols such as medication delivery (drop off)	2	5
Reproductive healthcare and birth control	6	15
Primary care needs (including managing co-morbidities)	9	22.5
Mental health needs	6	15
Chronic pain needs	4	10
Perinatal care	4	10
Vaccinations (including for COVID-Yes9)	1	2.5
Meeting non-clinical needs		
Social worker	10	25
Psychosocial assessment	3	7.5
Housing services	6	15
Mail services	1	2.5
Police-assisted diversion (PAD)	1	2.5
Food, clothing, hygiene	1	2.5
Transport	2	5
Engagement		
Outreach	9	22.5
Patient navigators	3	7.5
Mobile services (harm reduction, primary care, BBV, etc)	5	12.5
Drop-in service	4	10
Peer support	8	20
Shared decision making	5	12.5
Round the clock emergency support	3	7.5
Non-MOUD therapy		
Psychosocial/behavioral therapy	7	17.5
Virtual group or individual therapy	5	12.5
Mutual aid	2	5
Counseling	5	12.5

Ten (10/33; 27%) of the US services identified in this review were national or systems-wide services, delivering care across state boundaries or across a large geographic area within a state. This included the Veterans Health Administration,^42,43^ the NYC Health + Hospitals system,^44^ and the Los Angeles County Department of Health Services.^45^ State-wide services covering South Carolina^46^ and Pennsylvania^47,48^ were also represented. Two of the nationwide services were virtual only providers, one available in 30 US states^49^ and the other in 14 US states.^50^

All seven non-US services were national or systems-wide providers. Specifically, the Irish national model of remote care for opioid agonist treatment^51,52^ and the Norwegian national OUD treatment services^53^ were represented in this review. Two of the three Canadian services covered the province of Ontario.^54,55^ The third identified Canadian service covered the province of Alberta and is the only non-US example of a virtual only TMOUD service.^56^ One service provided care for OUD across a semi-rural county in England,^57,58^ and one is a publicly funded addiction treatment and research center catering to several northern and western states of India.^59^

All 40 services offered sublingual buprenorphine as a MOUD option. Among the US services in this review, only the NYC Health + Hospitals system offered methadone through four hospital-based Opioid Treatment Programs (OTPs).^44^ These OTPs did not provide telemedicine for patients already prescribed/taking methadone, despite the US regulatory changes allowing this provision.^44^ All 33 US services in this review offered buprenorphine via telemedicine, dubbed tele-buprenorphine in some articles.^60–62^ A small number of patients from services in Pennsylvania were offered buprenorphine depot injection.^47^ Among the seven non-US services, the Drug De-addiction and Treatment Center, in Chandigarh, India, was unable to provide MOUD without face-to-face contact with patients due to national regulatory restrictions on the prescribing of controlled drugs.^59^ The remaining six non-US services offered both buprenorphine and methadone via telemedicine.

In Ireland, England, and Ontario, the tendency has been for methadone prescribing to be more common than buprenorphine.^63–65^ The Virtual Opioid Dependency Program (VODP) in Alberta, Canada, did not offer patients induction to methadone in its pilot phase in 2017 but did introduce this from 2018 onwards.^56^ This makes VODP the only fully virtual TMOUD service in this review offering both buprenorphine and methadone inductions.^56^ In Norway, as of 2021, 75% of patients prescribed with MOUD were provided predominantly sublingual buprenorphine, and increasingly since 2019, buprenorphine depot injection.^66^ Indeed, 15% of patients on MOUD in Norway were provided monthly buprenorphine depot injections.^66^

### Telemedicine-specific challenges and solutions

 Table 3 describes key telemedicine delivery processes undertaken by the services in this review. More than half the services (23/40; 57.5%) were fully hybridized, offering virtual and in-person access to both clinical and non-clinical interventions. Seven of these services allowed patients to determine for themselves whether they wished to have virtual or in-person contact. A small number of services were more restricted in how they used telemedicine. For example, four services had fixed criteria for whom they permitted the use of telemedicine for, determined by measures of stability, according to threshold of care needs or an agreement to engage in a certain number or pattern of clinical contacts.^47,53,67,68^ Similarly, while more than half of the services here (21/40; 52.5%) permitted induction onto MOUD through telemedicine, a small number provided virtual care for patients on established treatment only (5/40; 12.5%).

**Table 3.** Telemedicine-specific service characteristics.

Characteristic	*N*	%
Telemedicine facilitator	18	45
Hybrid: All aspects	23	57.5
Hybrid: Non-prescriber only	6	15
Audio only	4	10
Audio and video by choice	12	30
Video only	5	12.5
Must have a rationale for telemedicine appointment	4	10
Patient may choose telemedicine or in person	7	17.5
Allows new patients (treatment induction)	21	52.5
Allows only patients stabilized on MOUD	2	5
Allows only patients already with the service	3	7.5
E-consultation to other hospital specialties or primary care	2	5.0
Clinical registries	1	2.5
Dedicated smartphone app	2	5
Chat channels to connect with support staff	2	5
Pharmacy finder tool (stock availability, proximity, non-stigmatizing service)	1	2.5
After hours troubleshooting line	2	5
Evidence-based buprenorphine induction tool (mobile app)	1	2.5
Toxicology screening process		
Clinically indicated only	14	35
Contingency management	6	15
Mandatory random testing	5	12.5
Asynchronous sample collection	4	10
Novel/technological support	3	7.5
Waived	3	7.5

Almost half the services (18/40; 45%) provided telehealth facilitation to support engagement with TMOUD consultations, and bridge potential digital literacy or access issues. In recognition that many patients were unable to afford a smartphone or internet connectivity, 16 services offered an audio option (16/40; 40%), making telemedicine accessible to those with access to a standard handset or fixed line phone.

A key issue in providing TMOUD has been how near patient drug testing was to occur. Drug testing, to aid in OUD diagnosis, screening for polysubstance use, monitoring progress and stability, and confirming compliance with or detecting potential diversion of medication, is typically considered standard practice by treatment services.^52,69–71^ While guidance were produced to ensure that drug testing did not become a barrier to accessing MOUD during the pandemic,^70,72^ many services continued to place an arguably excessive importance in retaining this practice. Fourteen services (14/40; 35%) expected patients to provide samples for drug testing where clinically indicated for example if there were concerns around stability or diversion of MOUD. Five services (5/40; 12.5%) continued with mandatory drug testing as part of a formal treatment program, and six (6/40; 15%) used drug testing to support contingency management decisions where proof of abstinence would result in more entitlement to take-home doses of MOUD. Three services provided novel drug testing approaches that included posting out urine kits to the patient's home so a drug test could be carried out on demand during a virtual consult,^49^ or the use of saliva-based drug testing kits where the clinician observed the test being taken live during a video consult.^47^

Finally, the extended capabilities of a digital medium were capitalized upon by small number of services that provided dedicated apps to manage appointments and consults,^17,49^ chatroom channels to interact with support staff,^17,49^ a pharmacy finder with stock checking and stigma rating functionality,^49^ a mobile phone-based buprenorphine induction tool,^49^ and a digital overdose response system.^56^

### Defining TMOUD models of care

Through our analysis of the 40 services identified in this review, detailed in the Supplemental Tables 2–5, we have defined four TMOUD models of care that are described in this section. Table 4 provides a summary of each care model described below. These are TMOUD provided in the context of improving health inclusion, facilitating transitions through care, supporting complex care needs, and increasing service resilience. The models of care described here are not mutually exclusive and do have overlapping characteristics.

**Table 4.** Summary of TMOUD care models.

TMOUD model	Description
TM 1.0 Inclusion health focus.	*Definition.* The model is focused on reaching marginalized, easily ignored groups at the intersections of multiple forms of exclusion including race, homelessness, criminal justice involvement, and forced displacement. *Key aspects of care* include outreach through street medicine/mobile clinics, healthcare facilitators, harm reduction workers, telemedicine facilitators, and multi-component care. *Features:* *TM 1.1 Places of safety.* Care is brought to the patient where they are and feel safe. *TM 1.2 Trauma-informed.* The service is guided by trauma-informed principles, culturally appropriate, peer-supported care. *TM 1.3 Low threshold.* Provides low threshold, no wrong door, low-barrier entry into treatment. *TM 1.4 Social determinants of health*. The service is sensitive to social determinants of health including digital exclusion and mitigates against these.
TM 2.0 Transitions through care	*Definition.* The model is focused on promoting the patient's social stability and continuity of care across jurisdictional boundaries and points of transition where overdose risk may be magnified. *Key aspects of care* include bridge clinics, shared care arrangements between specialist and primary care, and multidisciplinary services such as joint OUD and perinatal services. *Features:* *TM 2.1 Patient journey mapping.* Identification points of high risk of patient attrition, for example, leaving prison, discharge from hospital, emergency department, or treatment service, becoming homeless or relocating. *TM 2.2 Process mapping and risk mitigation*. Removes barriers to accessing a MOUD prescription, clinical review, or MOUD supply through a dispensing pharmacy. *TM 2.3 Chronic condition management*. Incorporates principles of managing a chronic relapsing remitting condition where longitudinal continuity of care reduces the risk of adverse health outcomes. *TM 2.4 Multiple episodes of care.* Recognizing, accepting, and allowing multiple episodes of care across the condition.
TM 3.0 Complexity of care needs	*Definition.* The model is designed around multiple unmet care needs of including housing, legal and income support, treatment of co-morbid physical and mental health conditions, and harm reduction interventions to prevent blood-borne virus transmission, soft tissue infections, and overdose deaths. *Key aspects of care* include care navigators or medical case managers to coordinate interactions with health services, navigate complex bureaucratic systems, create individualized care plans, and support them in meeting their goals. *Features:* *TM 3.1 Multidisciplinary and multisectoral care* incorporating social workers, counselors, nurses, primary care physicians, case managers, legal advisors, and outreach workers. *TM 3.2 Flexibility to tailor interventions*/programs to different needs for example services for people with HIV or HCV, inclusion health primary care, or street medicine services. *TM 3.3 Education and empowerment* of patients through peers or culturally appropriate workers to facilitate self-advocacy.
TM 4.0 Increasing service resilience and quality	*Definition.* The model is designed around an existing service's needs to fill resource or geographic gaps, remove administrative or clinical burden, introduce cost efficiencies, or to increase flexibility to innovate, change, adapt, or survive. *Key aspects of care* are determined at organizational level with defined goals, criteria, boundaries, and processes, and there may be limited opportunities for service-users involvement in its design and delivery. *Features:* *TM 4.1 Internally or externally sourced.* TMOUD pathways and expertise are developed internally or brought in from an external vendor. *TM 4.2 Pre-defined intake criteria.* The TMOUD service has specific intake criteria. Where a TMOUD vendor is used, intake may rely on shared care protocols with the host organization to ensure that an appropriate screening process has occurred before referral. *TM 4.3 Co-produced clinical pathways.* Where a vendor is used, service delivery pathways will need to be co-produced with the host organization to fit with the organization's strategic and pragmatic aims in introducing TMOUD.

### TMOUD within inclusion health focused services

Inclusion health target populations refer to people who are disproportionately affected by inequalities in the social determinants of health, due to intersecting experiences of marginalization from homelessness, forced displacement, sex work, or criminal justice involvement, frequently underpinned by problematic drug and alcohol use.^11,73^ Among inclusion health populations, the mortality rate is almost 8 times the male average and nearly 12 times the female average, significantly greater than accounted for by the social gradient in health.^74^ Twelve of the services in this review (12/40; 30%) had an inclusion health focus targeting easily ignored groups such as people experiencing homelessness,^45,75–82^ involved with criminal justice^75,79,80,83^ or with sex work.^84^

These services offered a low threshold (minimal barrier) for entry typically characterized by same-day treatment entry^45,51,52,56,60–62,76–81,85–87^ and permitting treatment induction at home rather than exclusively at a dispensing pharmacy.^45,49,56,60,61,76–81,86,87^ In keeping with trauma-informed principles in providing care to people who use drugs (PWUD),^88^ a non-judgmental attitude respectful of the patients goals in engagement with treatment were prioritized,^45,49,56,60,61,76–81,86,87^ including a reduction in illicit substance use as opposed to a commitment to abstinence.^56,60,75–77,79,81,87^

The cornerstone of this model involved making OUD care available in non-traditional sites, where the patient felt safe and more likely to attend. This is demonstrated by the provision of TMOUD at SSPs, which are community-based interventions offering sterile injecting equipment and blood-borne virus preventative strategies such as testing and treatment.^89^ Eight (8/40; 20%) of the services in this review provided TMOUD via SSPs,^75–80,82,84,87,90^ and three also worked through overlapping harm reduction services.^75,84,85^ Barriers were broken down further by delivering TMOUD directly to people on the street. Examples of this have included the Los Angeles County Department of Health Services low-barrier telephone service^45^ that used community health workers entering homelessness encampments and linking patients directly with providers, or volunteer delivered foot or bicycle-based street outreach through the Harm Reduction and BRidges to Care (HRBR) Clinic, in Portland, Oregon.^90^

A further key characteristic of this TMOUD model was a nuanced perspective on digital exclusion.^75^ For example, Aronowitz et al., highlighted that the remote clinician's attitude toward risk, expectations, and measures of patient stability and consequent judgment on suitability for telemedicine consults ran counter to in-person observations of non-clinical support workers in SSPs.^75^ In this context, digital exclusion was not caused by a lack of access to technology, but to clinician discomfort in the use of this technology for a specific patient group.^75^ Other examples of a nuanced understanding of digital exclusion was demonstrated by the Infectious Disease Elimination Act (IDEA) Syringe Services Program (SSP) in Miami, Florida,^76^ and the associated Telehealth Solution for HIV and Addiction-Related Problems among People who inject drugs (T-SHARP)^77,78,81^ research project. Specifically, while the telemedicine consult was a critical element of care, there were several other essential components needed to translate it into successful engagement in MOUD. The IDEA SSP service for example invested in the provision of flexible medication management protocols that allowed for fixed location lockers or street-based medication drop-offs to ensure that people experiencing homelessness were able to safeguard their prescription medications supply.^77^ Similarly, the STAMINA study at three SSP sites in Chicago, Illinois, incorporated transport for patients to attend the pharmacy to obtain their supply of buprenorphine following a telemedicine consult, or to access an OTP, should they opt for methadone as their MOUD option.^87^

Finally, digital exclusion cannot be divorced from the individual's overall socioeconomic deprivation and disadvantage.^91^ In other words, where the individual is unable to meet housing, financial, and nutritional needs, it is unlikely for them to be concerned about owning and maintaining a digital device to utilize telemedicine. Examples to mitigate against this included the telephone booth model of TMOUD by the Centre for Harm Reduction, Homeless Health Care Los Angeles.^82^ Further, several services providing TMOUD also extended supports to address social determinants of health including housing,^75,77,81,82,92,93^ food, clothing, and hygiene.^82^

Several services recognized the importance of building trust and creating a sense of psychological safety among PWOUD in need of treatment through the use of peer outreach workers.^49,60,75–77,79–81,84,92^ Nevertheless, there was evidence to show that Latinx and Black PWUD were less likely than White PWUD to access SSPs, harm reduction interventions, and OUD services more generally.^94^ Indeed, this was the reflection of the Los Angeles County Department of Health Services low-barrier telephone service that continued to find disparities in Black and Latinx populations access of MOUD despite their healthcare worker outreach approach.^45^ One service, the Honoring Individual Power & Strength (HIPS) clinic, in Washington, DC, bucked the trend with predominantly Black (90.6%), male (74.4%) clients with a mean age of 53 years old. Most HIPS clinic staff originated from the same community and shared lived experiences with clients, including recovery from substance use, which contributed to the effectiveness of their TMOUD model in addressing racial inequalities to access.^84^

### TMOUD facilitating transitions through care

A key aspect of the provision of MOUD is continuity through key periods of transition across or between services and jurisdictions.^19^ Examples of key transition points include people entering or leaving the criminal justice system,^19^ leaving an emergency department following a non-fatal opioid overdose,^29,95^ moving from specialist treatment programs to community-based maintenance services,^96^ leaving an inpatient hospital ward on completion of medical treatment,^19^ or an unplanned departure from an OTP for non-adherence to a treatment plan.^97^ These periods of transition represent key touchpoints where substance use-related harms are more likely to occur, and overlap with opportunities to modify this risk.^19^

Ten services in this review described bridge clinic models designed specifically to provide low-barrier, transitional MOUD services to reduce the increased risk associated with periods of care transition.^17,47,56,60–62,85,86,90^ These services became particularly important with the onset of the COVID-19 pandemic due to policies of rapid reductions in prison populations, resulting in a large number of people leaving incarceration with urgent MOUD needs.^98^ Six of these 10 TMOUD bridge services provided care to people leaving emergency departments.^17,47,60,85^ An emergency department in Rhode Island conducted a callback pilot to extend the reach of the bridge model of care, contacting people who suffered a non-fatal opioid overdose for a follow up telephone call on discharge, offering harm reduction resources and access to TMOUD.^60^ The Faster Paths Low-barrier bridge clinic offered through The Grayken Center for Addiction at the Boston Medical Center not only provided care to patients identified through the emergency department, but also permitted direct walk-in access for patients.^85^ The NYC Public Hospital System Low-Threshold Tele-Buprenorphine bridge clinic was able to provide care to patients referred by hospital and clinic staff, correctional health services, community organizations supporting reentry, homeless shelters, social service, community organizations, and harm reduction programs, as well as self-referrals from patients hearing about the service by word of mouth and online searches.^61^

In addition to the bridge clinic model, this review identified two services designed to offer specialist transitional telemedicine care to women with OUD and going through pregnancy.^68,93^ These services were particularly important to maximize the engagement with both perinatal and OUD care, both of which improve health and social outcomes before, during, and after pregnancy.^68,93^ Five shared care models through telemedicine were identified, where specialty services like addiction psychiatry carried out diagnosis, assessment, MOUD induction, and handed off to community-based services or primary care for longer term chronic care management.^25,42,43,46,51–53,62,99,100^

The VODP in Alberta, Canada, exemplified the full potential of telemedicine in delivering treatment continuity through periods of transition and increased risk.^56^ VODP was fully integrated into the Alberta Health Service and used the province-wide telehealth network and health insurance.^56^ This enabled access to TMOUD to any qualifying individual, practitioner, or service (including criminal justice, harm reduction, and hospitals) in Alberta through a single toll free number.^56^ Reciprocally, a broad range of Alberta-wide community-based resources such as laboratories for urine drug screening, pharmacies for observed MOUD dosing, and unscheduled care telephone lines were available to VODP staff and patients.^56^

### TMOUD supporting complex care needs

Several TMOUD services identified in this review evolved to address the complex health and social care needs of PWOUD. Examples included those providing care for people with or at risk of HIV,^75–81^ HCV,^79,80,92^ multi-morbid health conditions best addressed by primary or generalist care^42,43,47,48,61,62,85,100^ including through street medicine,^79^ and co-morbid mental health conditions.^55,71^ The provision of care navigators to help patients to coordinate complex bureaucratic systems of health and social care was a key component in addressing complex care needs.^45,61,62,92^ Embedding TMOUD within primary care allowed for cultivation of long term, longitudinal relationships, and provision of resources and expertise to manage chronic co-morbid medical conditions including chronic pain and cardiovascular and respiratory disease, another critical feature of this model.^42,43,47,51,62,66,85,100^

Other key needs addressed by services supporting patients with complex needs included the provision of onsite phlebotomy,^78,79^ hepatitis C testing and cure,^75,78,79,85^ HIV testing, and antiretroviral therapy for suppression,^78,79,85^ wound care including incision and drainage,^75–78,82,85^ and reproductive care needs.^68,75–78,85^ Mental health needs were met through telepsychiatry, virtual psychology or counseling, and mobile app-based chatrooms.^47,49,55,68,71,76–78,85,93^ In order to educate and empower patients to prioritize their own health needs and goals, some services prioritized shared decision making to develop individualized care plans.^17,75,78,93^

Some services provided access to crisis care through stabilization units to support people in acute withdrawals or intoxication,^17^ or through round the clock telephone or app-based emergency support.^50,56,68^ New avenues of meeting complex health needs were made possible in the Substance Treatment and Recovery (STAR) clinic in South Carolina, following COVID-19-related transitioning to TMOUD, where a new range of cross-specialty and cross-disciplinary e-consults were introduced.^46^ The Norwegian national OUD treatment service identified improvements in liaising with non-medical colleagues in social and housing services through multidisciplinary case management facilitated by telemedicine.^53^

### TMOUD to increase service resilience

The term “TMOUD vendors” has been used to describe the outsourcing of MOUD provision to specific telemedicine providers to expand community-based access to this form of treatment.^101^ Hser et al., described a feasibility study looking at the use of TMOUD vendors to expand MOUD availability to rural primary care services for example. Participating rural practices screened for OUD and directed appropriate patients to a telemedicine consult for buprenorphine induction and stabilization before taking on their longer term management.^101^ Both Bicycle Health^49^ and Ophelia Health^50^ offered opportunities to work with health systems and providers to offer tele-buprenorphine to patients meeting pre-determined criteria. Other examples included the STAMINA study at three SSP sites in Chicago, where a national TMOUD vendor remotely assessed potential patients and either inducted suitable patients unto buprenorphine or facilitated their attendance at an OTP that offered methadone.^87^ Critically, the intake criteria TMOUD vendors used had the potential of excluding patients with complex needs or those perceived as having significant social or clinical instability.^50^

The VODP service, fully integrated into the existing healthcare infrastructure in Alberta, Canada,^56^ supported mainstream community OUD service resilience through the provision of flexible continuity of care. Indeed, a fully integrated virtual model offering seamless transitions through care may ultimately contribute to overall treatment system resilience by reducing demand on traditional services to respond to acute crises and high-risk situations among PWOUD. VODP provided bridging MOUD services, allowed for repeated treatment episodes, and was able to provide ongoing medical case management if there is no local capacity to take over patient care.^56^ Similarly, the US Veteran Health Administration funded regional tele-mental health hubs, which facilitated the sharing of clinical cover to less well-resourced areas, a facility that was adapted to deliver TMOUD during the pandemic.^43^

The effectiveness of this model is contingent on the TMOUD vendor understanding and engaging with the system and organizational needs of the host service,^50,101^ the community, and individual patient care needs.^49^ For example, one vendor supporting a high threshold abstinence focused rural service reflected on the disadvantages in being unable to influence other critical programmatic components such as the patient discharge policy and obligations to attend counseling.^102,103^ Additionally, fully virtual TMOUD services in this review were not explicit in how key harm reduction services such as take-home naloxone, overdose awareness and response training, sterile injecting equipment, and fentanyl test strips were to be provided.^87,104^ The exception was VODP that provided access to an Alberta-wide app-based digital overdose response system.^56^ Exclusively, virtual services may need to be increasingly intentional, proactive, and innovative in ensuring that high quality harm reduction interventions are incorporated into their delivery of TMOUD.

## Discussion

To our knowledge, this systematic scoping review is the first exploration of the international peer-reviewed literature to identify models of TMOUD delivery. Through the identification and characterization of discrete services within our data set, we have been able to empirically define four over-arching models of TMOUD. Three of these models addressed some of the complexities inherent in providing care to PWOUD, for example, the need to actively reach marginalized individuals, to minimize breaks in MOUD provision during periods of transition, and to opportunistically address holistic care needs beyond OUD. The final TMOUD model of care, arguably the dominant focus of post-pandemic policy discussions, related to improving and preserving service resiliency and efficiency.

By defining these four overlapping models of care, we hope to contribute to a more nuanced discussion on the policy challenges involved in designing telemedicine for PWOUD. This has relevance for healthcare jurisdictions examining alternative reimbursement models such as bundled payments (expected costs for a defined episode of care) as opposed to fee per service or capitation (a standard fee per patient regardless of services provided) models.^105^ Specifically, the TMOUD models described here provide context to define clinical episodes of care matched to the needs of PWOUD. By defining what these PWOUD-focused episodes of care look like, we also hope to influence the quality standards of this care and future outcome and evaluation measures.

Further, alternative payment models that allows the flexible use of in-person and telemedicine tools^106^ to fit specific functional deliverables, for example, inclusion health, care transitions, complex co-morbidities, and service resilience, may lead to innovative approaches that reach marginalized groups, rather than being accessible only to those with a higher level of health cover or digital competence. Such an approach may mitigate against exclusively virtual TMOUD services cherry-picking higher-margin patients, leaving hybrid or traditional providers grappling with increased health inequity and negative financial impacts.^41^

The importance of a nuanced understanding of digital exclusion when applying TMOUD models of care has been highlighted in this review. For example, there is currently a debate on whether audio only telemedicine should be reimbursed in the same way as video consultations,^106^ with policymakers leaning toward disallowing this in the longer term. This is partly due to concerns that audio only services will result in increased costs without improvements in efficiency or workload. Nevertheless, some of the more successful TMOUD services identified in this review, which have engaged people at the extreme margins of society, have been audio only services.^45,84^ It is possible therefore that current thinking that devalues audio only telemedicine may contribute to worsening digital exclusion. The growing evidence base for audio only TMOUD in populations most vulnerable to digital exclusion may serve to temper this debate^107^ and lead the way toward future prospective randomized controlled trials for this treatment modality.

Another concern is that digital exclusion may become conflated with other structural causes of social exclusion and inequality. More specifically, the reasons why some TMOUD services were not successful in attracting Black and Latinx PWOUD may be connected to an entrenched mistrust of authorities and services rather than due to a lack of digital competence or resources.^94^ Strategies to address digital exclusion that focuses on access to technology or improving digital literacy alone^34^ may therefore miss the mark and fail to engage these groups in TMOUD. A particular challenge lies in identifying how cultural competence, trust building, and breaking down barriers caused by structural violence may be embedded within telemedicine approaches offered to marginalized and oppressed communities.

The main limitation of this review is that it is dominated by US studies, which may impact on its applicability to other countries and health systems. Further, as TMOUD in the US has mainly provided sublingual buprenorphine, information is limited on how methadone or injectable depot buprenorphine may be provided through telemedicine. This gap does need to be addressed considering the lower retention in treatment of patients on buprenorphine (46%) compared to methadone (74%),^108^ and the different safety concerns relating to the two medications.^70^ Both Irish^52^ and Canadian^70^ guidance on TMOUD provide detail on how methadone inductions and maintenance can be conducted, and risk managed, and future research should consider more diverse use of different medication options. Additionally, concerns over medication diversion, the need for frequent pharmacy attendance, and problems with identifying pharmacies to dispense buprenorphine may be addressed by exploring the more extensive use of injectable depot buprenorphine, as was done in the national Norwegian OUD service.^53^

Finally, both in the US, and internationally, there are likely to be private and public entities that have carried out intentional or ad hoc TMOUD activities that will not have been captured by this review. There is therefore a need to understand the lack of peer-reviewed literature on TMOUD from other countries and within that, from treatment providers not typically captured by conventional research approaches. The UK, for example, is said to have introduced telemedicine to facilitate more widespread induction of patients to MOUD in response to the pandemic,^109,110^ yet there is limited peer-reviewed or grey literature to verify this claim, or to support local best practice guidance on practice and implementation of TMOUD.

## Conclusion

In this review, we have detailed how services providing OUD care have incorporated telemedicine to facilitate the provision of medications such as methadone or buprenorphine. Specifically, we have characterized four models of TMOUD that includes its use to facilitate inclusion health, transitions through care, the management of complex care needs, and maintaining the resilience of in-person high acuity OUD services. This characterization supports a more nuanced discussion around the role of telemedicine in delivering care for PWOUD, with implications for how such services are funded and avoiding the risk of worsening digital exclusion among already marginalized and vulnerable groups. Further, our characterization of TMOUD models may guide the development of service evaluation metrics that link more directly to the different but overlapping constellations of PWOUD need. This may lead to incentivizing the retention and strengthening of TMOUD programmatic components that breaks down structural barriers to treatment access for PWOUD.

## Supplemental Material

sj-pdf-1-jtt-10.1177_1357633X231195607 - Supplemental material for Understanding the use of telemedicine across different opioid use disorder treatment models: A scoping review Supplemental material, sj-pdf-1-jtt-10.1177_1357633X231195607 for Understanding the use of telemedicine across different opioid use disorder treatment models: A scoping review by Joseph Tay Wee Teck, Jenna L Butner, and Alex Baldacchino in Journal of Telemedicine and Telecare

## References

[1] American Psychiatric Association D, Association AP. Diagnostic and statistical manual of mental disorders: DSM-5. Washington, DC: American Psychiatric Association, 2013.

[2] UNODC. *World Drug Report: Executive summary and policy implications*. Vienna: United Nations Office on Drugs and Crime, https://www.unodc.org/res/wdr2022/MS/WDR22_Booklet_1.pdf (2022, accessed 26 January 2023).

[3] Alexander K Pogorzelska-Maziarz M Gerolamo A , et al. The impact of COVID-19 on healthcare delivery for people who use opioids: A scoping review. Subst Abuse Treat Prev Policy 2021; 16: 60. 34372900 10.1186/s13011-021-00395-6 PMC8352141

[4] Czeisler MÉ . Mental health, substance use, and suicidal ideation during the COVID-19 pandemic — United States, June 24–30, 2020. MMWR Morb Mortal Wkly Rep 2020; 69: 1049–1057. DOI: 10.15585/mmwr.mm6932a1. PMC7440121 32790653

[5] MacKinnon L Socías ME Bardwell G . COVID-19 and overdose prevention: Challenges and opportunities for clinical practice in housing settings. J Subst Abuse Treat 2020; 119: 108153. 33032862 10.1016/j.jsat.2020.108153 PMC7532988

[6] McGrail K Morgan J Siddiqi A . Looking back and moving forward: Addressing health inequities after COVID-19. The Lancet Regional Health - Americas 2022; 9: 100232. 35313508 10.1016/j.lana.2022.100232 PMC8928332

[7] Volkow ND . Collision of the COVID-19 and addiction epidemics. Ann Intern Med 2020; 173: 61–62. 32240293 10.7326/M20-1212 PMC7138334

[8] Wang J . Examining Low-Barrier Buprenorphine Treatment during COVID-19 for Individuals Experiencing Housing Insecurity and Homelessness, https://dataspace.princeton.edu/handle/88435/dsp01n583xz11f (2021, accessed 25 March 2023).

[9] Bambra C Riordan R Ford J , et al. The COVID-19 pandemic and health inequalities. J Epidemiol Community Health 2020; 74: 964–968. 32535550 10.1136/jech-2020-214401 PMC7298201

[10] Wachtler B Michalski N Nowossadeck E , et al. Socioeconomic inequalities and COVID-19 – A review of the current international literature. J Health Monit 2020; 5: 3–17. 10.25646/7059 PMC8734114 35146298

[11] Marmot M . Inclusion health: Addressing the causes of the causes. Lancet 2018; 391: 186–188. 29137870 10.1016/S0140-6736(17)32848-9

[12] Marmot M Allen J . COVID-19: Exposing and amplifying inequalities. J Epidemiol Community Health 2020; 74: 681–682. 32669357 10.1136/jech-2020-214720 PMC7577092

[13] Hailu R Mehrotra A Huskamp HA , et al. Telemedicine use and quality of opioid use disorder treatment in the US during the COVID-19 pandemic. JAMA Network Open 2023; 6: e2252381. 10.1001/jamanetworkopen.2022.52381 PMC10038015 36692880

[14] Wang L Weiss J Ryan EB , et al. Telemedicine increases access to buprenorphine initiation during the COVID-19 pandemic. J Subst Abuse Treat 2021; 124: 108272. 33771276 10.1016/j.jsat.2020.108272 PMC7833481

[15] Haley DF Saitz R . The opioid epidemic during the COVID-19 pandemic. JAMA 2020; 324: 1615–1617. 32945831 10.1001/jama.2020.18543

[16] Radfar SR De Jong CAJ Farhoudian A , et al. Reorganization of substance use treatment and harm reduction services during the COVID-19 pandemic: A Global Survey. Front Psychiatry 2021; 12, Epub ahead of print 2021. DOI: 10.3389/fpsyt.2021.639393. PMC8135096 34025471

[17] Gainer DM Wong C Embree JA , et al. Effects of telehealth on dropout and retention in care among treatment-seeking individuals with substance use disorder: A retrospective cohort study. Subst Use Misuse 2023; 58: 481–490. 36710568 10.1080/10826084.2023.2167496

[18] Czeisler MÉ . A case for permanent adoption of expanded telehealth services and prescribing flexibilities for opioid use disorder: Insights from pandemic-prompted emergency authorities. JAMA Psychiatry 2022; 79: 950–952. 36044204 10.1001/jamapsychiatry.2022.2032

[19] Strang J Volkow ND Degenhardt L , et al. Opioid use disorder. Nat Rev Dis Primers 2020; 6: 1–28. 31919349 10.1038/s41572-019-0137-5

[20] Bell J Strang J . Medication treatment of opioid use disorder. Biol Psychiatry 2020; 87: 82–88. 31420089 10.1016/j.biopsych.2019.06.020

[21] Calcaterra SL Bach P Chadi A , et al. Methadone matters: What the United States can learn from the global effort to treat opioid addiction. J GEN INTERN MED 2019; 34: 1039–1042. 30729416 10.1007/s11606-018-4801-3 PMC6544670

[22] Cheetham A Picco L Barnett A , et al. The impact of stigma on people with opioid use disorder, opioid treatment, and policy. Subst Abuse Rehabil 2022; 13: 1–12. 35115860 10.2147/SAR.S304566 PMC8800858

[23] Volkow ND . Medications for opioid use disorder: Bridging the gap in care. Lancet 2018; 391: 285–287. 29150199 10.1016/S0140-6736(17)32893-3

[24] Volkow ND Icaza MEM-M Poznyak V , et al. Addressing the opioid crisis globally. World Psychiatry 2019; 18: 231–232. 31059614 10.1002/wps.20633 PMC6502427

[25] Chan B Bougatsos C Priest KC , et al. Opioid treatment programs, telemedicine and COVID-19: A scoping review. Subst Abus 2022; 43: 539–546. 34520702 10.1080/08897077.2021.1967836 PMC11970435

[26] Patton T Revill P Sculpher M , et al. Using economic evaluation to inform responses to the opioid epidemic in the United States: Challenges and suggestions for future research. Subst Use Misuse 2022; 57: 815–821. 35157549 10.1080/10826084.2022.2026969 PMC8969147

[27] Lowrie R McPherson A Mair FS , et al. Baseline characteristics of people experiencing homelessness with a recent drug overdose in the PHOENIx pilot randomised controlled trial. Harm Reduct J 2023; 20: 46. 37016418 10.1186/s12954-023-00771-4 PMC10071267

[28] Hedden L McCracken RK Spencer S , et al. Advancing virtual primary care for people with opioid use disorder (VPC OUD): A mixed-methods study protocol. BMJ Open 2022; 12: e067608. 10.1136/bmjopen-2022-067608 PMC9516147 36167365

[29] Hawk K Hoppe J Ketcham E , et al. Consensus recommendations on the treatment of opioid use disorder in the emergency department. Ann Emerg Med 2021; 78: 434–442. 34172303 10.1016/j.annemergmed.2021.04.023

[30] Korownyk C Perry D Ton J , et al. Managing opioid use disorder in primary care: PEER simplified guideline. Canadian Family Physician 2019; 65: 321–330. 31088869 PMC6516701

[31] Eaves ER Trotter RTI Baldwin JA . Another silver lining?: Anthropological perspectives on the promise and practice of relaxed restrictions for telemedicine and medication-assisted treatment in the context of COVID-19. Hum Organ 2020; 79: 292–303. 33551465 10.17730/1938-3525-79.4.292 PMC7861509

[32] Krawczyk N Allen ST Schneider KE , et al. Intersecting substance use treatment and harm reduction services: Exploring the characteristics and service needs of a community-based sample of people who use drugs. Harm Reduct J 2022; 19: 95. 36002850 10.1186/s12954-022-00676-8 PMC9400571

[33] Teck JTW Zlatkute G Perez A , et al. Key implementation factors in telemedicine-delivered medications for opioid use disorder: A scoping review informed by normalisation process theory. The Lancet Psychiatry 2023; 10: 50–64. 36526346 10.1016/S2215-0366(22)00374-1

[34] Uscher-Pines L Riedel LE Mehrotra A , et al. Many clinicians implement digital equity strategies to treat opioid use disorder. Health Aff 2023; 42: 182–186. 10.1377/hlthaff.2022.00803 PMC10186211 36745832

[35] Uscher-Pines L Sousa J Raja P , et al. Treatment of opioid use disorder during COVID-19: Experiences of clinicians transitioning to telemedicine. J Subst Abuse Treat 2020; 118: N.PAG-N.PAG. 10.1016/j.jsat.2020.108124 PMC7456454 32893047

[36] Uscher-Pines L Raja P Mehrotra A , et al. Health center implementation of telemedicine for opioid use disorders: A qualitative assessment of adopters and nonadopters. J Subst Abuse Treat 2020; 115: 108037. 32600625 10.1016/j.jsat.2020.108037 PMC7327134

[37] Andraka-Christou B . Addressing racial and ethnic disparities in the use of medications for opioid use disorder. Health Affairs (Project Hope) 2021; 40: 920–927. 34097509 10.1377/hlthaff.2020.02261

[38] Peters MD Godfrey CM McInerney P , et al. The Joanna Briggs Institute reviewers’ manual 2015: methodology for JBI scoping reviews.

[39] Tricco AC Lillie E Zarin W , et al. PRISMA extension for scoping reviews (PRISMA-ScR): Checklist and explanation. Ann Intern Med 2018; 169: 467–473. 30178033 10.7326/M18-0850

[40] Lagisetty P Klasa K Bush C , et al. Primary care models for treating opioid use disorders: What actually works? A systematic review. PLOS ONE 2017; 12: e0186315. 10.1371/journal.pone.0186315 PMC5645096 29040331

[41] Tang M Chernew ME Mehrotra A . How emerging telehealth models challenge policymaking. Milbank Quarterly 2022; 100: 650–672. 36169169 10.1111/1468-0009.12584 PMC9576237

[42] Mattocks KM Moore DT Wischik DL , et al. Understanding opportunities and challenges with telemedicine-delivered buprenorphine during the COVID-19 pandemic. J Subst Abuse Treat 2022; 139: 108777. 35346533 10.1016/j.jsat.2022.108777 PMC8949846

[43] Brunet N Moore D Wischik D , et al. Increasing buprenorphine access for veterans with opioid use disorder in rural clinics using telemedicine. Subst Abus 2020; 43: 1–8. 10.1080/08897077.2020.1728466 32078492

[44] Avalone L King C Popeo D , et al. Increased attendance during rapid implementation of telehealth for substance use disorders during COVID-19 at the largest public hospital system in the United States. Subst Use Misuse 2022; 57: 1322–1327. 35611875 10.1080/10826084.2022.2079140

[45] Kennedy AJ George JS Rossetti G , et al. Providing low-barrier addiction treatment via a telemedicine consultation service during the COVID-19 pandemic in Los Angeles, county: An assessment 1 year later. J Addict Med 2023; 17: e64. 10.1097/ADM.0000000000001034 PMC9897115 35839323

[46] Fiacco L Pearson BL Jordan R . Telemedicine works for treating substance use disorder: The STAR clinic experience during COVID-19. J Subst Abuse Treat 2021; 125: 108312. 34016299 10.1016/j.jsat.2021.108312 PMC8561322

[47] Poulsen MN Santoro W Scotti R , et al. Implementation of telemedicine delivery of medications for opioid use disorder in Pennsylvania treatment programs during COVID-19. J Addict Med 2023; 17: e110–e118. 10.1097/ADM.0000000000001079 PMC10022523 36129690

[48] Kaur J Mania I Tirupathi R , et al. Impact of telemedicine on retention in medications for opioid use disorder (MOUD) treatment with buprenorphine in the times of COVID-19 pandemic: A retrospective chart review. J Rural Mental Health 2022; 46: 75–81.

[49] Rollston R Gallogly W Hoffman L , et al. Collaborative, patient-centred care model that provides tech-enabled treatment of opioid use disorder via telehealth. BMJ Innov 2022; 8: 117–122. DOI: 10.1136/bmjinnov-2021-000816.

[50] Williams AR Aronowitz S Gallagher R , et al. A virtual-first telehealth treatment model for opioid use disorder. J GEN INTERN MED 2023; 38: 814–816. 10.1007/s11606-022-07955-x PMC9714767 36456841

[51] Crowley D Delargy I . A national model of remote care for assessing and providing opioid agonist treatment during the COVID-19 pandemic: A report. Harm Reduct J 2020; 17: 1–5. DOI: 10.1186/s12954-020-00394-z. PMC7366558 32680520

[52] Durand L Keenan E Boland F , et al. Consensus recommendations for opioid agonist treatment following the introduction of emergency clinical guidelines in Ireland during the COVID-19 pandemic: A national Delphi study. Int J Drug Policy 2022; 106: 103768. 35738029 10.1016/j.drugpo.2022.103768 PMC9212711

[53] McDonald R Bech AB Clausen T . Flexible delivery of opioid agonist treatment during COVID-19 in Norway: A cross-sectional survey of provider experiences. *BMC Health Serv Res*. Epub ahead of print 18 January 2023. DOI: 10.21203/rs.3.rs-2212348/v1. PMC10485985 37679751

[54] Eibl JK Gauthier G Pellegrini D , et al. The effectiveness of telemedicine-delivered opioid agonist therapy in a supervised clinical setting. Drug Alcohol Depend 2017; 176: 133–138. 28535455 10.1016/j.drugalcdep.2017.01.048

[55] LaBelle B Franklyn AM Nguyen VPKH , et al. Characterizing the use of telepsychiatry for patients with opioid use disorder and cooccurring mental health disorders in Ontario, Canada. Int J Telemed Appl 2018; 2018: e7937610. 10.1155/2018/7937610 PMC5828243 29610570

[56] Day N Wass M Smith K . Virtual opioid agonist treatment: Alberta’s virtual opioid dependency program and outcomes. Addict Sci Clin Pract 2022; 17: 40. 35902924 10.1186/s13722-022-00323-4 PMC9330968

[57] Mayet S Mccaw I Hashmani Z , et al. Patient experience of telemedicine in addictions. BJPsych Open 2021; 7: S269–S270.

[58] Mayet S Gledhill A McCaw I , et al. Telemedicine in addictions: Feasibility randomised controlled trial. Heroin Addict Relat Clin Prob 2023; 25: 27–36.

[59] Ghosh A Mahintamani T Subodh BN , et al. Telemedicine-assisted stepwise approach of service delivery for substance use disorders in India. Asian J Psychiatr 2021; 58: 102582. 33607350 10.1016/j.ajp.2021.102582 PMC9760420

[60] Wightman RS Jacka B Uber J , et al. Tele-buprenorphine for emergency department overdose visit follow up and treatment initiation. Am J Emerg Med 2021; 50: 409–412. 34481260 10.1016/j.ajem.2021.08.071 PMC8665045

[61] Tofighi B McNeely J Yang J , et al. Outcomes of a NYC public hospital system low-threshold tele-buprenorphine bridge clinic at 1 year. Subst Use Misuse 2022; 57: 1337–1340. 35481461 10.1080/10826084.2022.2069269 PMC10107046

[62] Tofighi B McNeely J Walzer D , et al. A telemedicine buprenorphine clinic to serve New York city: Initial evaluation of the NYC public hospital system’s initiative to expand treatment access during the COVID-19 pandemic. J Addict Med 2022; 16: e40–e43. 10.1097/ADM.0000000000000809 PMC8339146 33560696

[63] Public Health England. *Research and analysis United Kingdom drug situation 2019: summary (Updated 31 March 2021)*, https://www.gov.uk/government/publications/united-kingdom-drug-situation-focal-point-annual-report/uk-drug-situation-2019-summary (2019, accessed 21 April 2023).

[64] Van Hout MC Crowley D McBride A , et al. Optimising treatment in opioid dependency in primary care: Results from a national key stakeholder and expert focus group in Ireland. BMC Fam Pract 2018; 19: 103. 29960593 10.1186/s12875-018-0792-8 PMC6026515

[65] Gomes T McCormack D Bozinoff N , et al. Duration of use and outcomes among people with opioid use disorder initiating methadone and buprenorphine in Ontario: A population-based propensity-score matched cohort study. Addiction 2022; 117: 1972–1981. 35257434 10.1111/add.15862 PMC9313829

[66] MacDonald T Gallo AT Basso-Hulse G , et al. Outcomes of patients treated with low-dose flumazenil for benzodiazepine detoxification: A description of 26 participants. Drug Alcohol Depend 2022; 237: 109517. 35688053 10.1016/j.drugalcdep.2022.109517

[67] Cales RH Cales SC Shreffler J , et al. The COVID-19 pandemic and opioid use disorder: Expanding treatment with buprenorphine, and combining safety precautions with telehealth. J Subst Abuse Treat 2022; 133: 108543. DOI: 10.1016/j.jsat.2021.108543. PMC8233546 34210567

[68] Guille C Simpson AN Douglas E , et al. Treatment of opioid use disorder in pregnant women via telemedicine: A nonrandomized controlled trial. JAMA Netw Open 2020; 3: e1920177–e1920177. 10.1001/jamanetworkopen.2019.20177 PMC7042863 32003816

[69] Hermes. *Virtual MOUD Treatment: Virtual Point-of-Care Toxicology Testing to Accompany Virtual Medication Assisted Treatment for Opioid Use Disorder*. Clinical Trial Registration NCT05448118, https://clinicaltrials.gov/ct2/show/NCT05448118 (4 November 2022, accessed 21 November 2022).

[70] Bruneau J Rehm J Wild TC , et al. *Telemedicine Support for Addiction Services: National Rapid Guidance Document.* Montreal, Quebec.: Canadian Research Initiative in Substance Misuse, https://crism.ca/wp-content/uploads/2020/05/CRISM-National-Rapid-Guidance-Telemedicine-V1.pdf (2020, accessed 6 March 2023).

[71] Zheng W Nickasch M Lander L , et al. Treatment outcome comparison between telepsychiatry and face-to-face buprenorphine medication-assisted treatment for opioid use disorder: A 2-year retrospective data analysis. J Addict Med 2017; 11: 138–144. 28107210 10.1097/ADM.0000000000000287 PMC5354971

[72] American Society of Addiction Medicine. *Caring For Patients During The Covid-19 Pandemic: Adjusting Drug Testing Protocols*, https://downloads.asam.org/sitefinity-production-blobs/docs/default-source/guidelines/covid-19/11-adjusting-drug-testing-protocols_final.pdf?sfvrsn=5dba58c2_2 (2020, accessed 10 July 2023).

[73] Luchenski S Maguire N Aldridge RW , et al. What works in inclusion health: Overview of effective interventions for marginalised and excluded populations. Lancet 2018; 391: 266–280. 29137868 10.1016/S0140-6736(17)31959-1

[74] Aldridge RW Story A Hwang SW , et al. Morbidity and mortality in homeless individuals, prisoners, sex workers, and individuals with substance use disorders in high-income countries: A systematic review and meta-analysis. Lancet 2018; 391: 241–250. 29137869 10.1016/S0140-6736(17)31869-X PMC5803132

[75] Aronowitz SV Engel-Rebitzer E Dolan A , et al. Telehealth for opioid use disorder treatment in low-barrier clinic settings: An exploration of clinician and staff perspectives. Harm Reduct J 2021; 18: 119. 34823538 10.1186/s12954-021-00572-7 PMC8614631

[76] Castillo M Conte B Hinkes S , et al. Implementation of a medical student-run telemedicine program for medications for opioid use disorder during the COVID-19 pandemic. Harm Reduct J 2020; 17: 1–6. DOI: 10.1186/s12954-020-00438-4. PMC7671179 33203460

[77] Suarez E Bartholomew TS Plesons M , et al. Adaptation of the Tele-Harm Reduction intervention to promote initiation and retention in buprenorphine treatment among people who inject drugs: A retrospective cohort study. Ann Med 2023; 55: 733–743. 36856571 10.1080/07853890.2023.2182908 PMC9980015

[78] Tookes HE Oxner A Serota DP , et al. Project T-SHARP: Study protocol for a multi-site randomized controlled trial of tele-harm reduction for people with HIV who inject drugs. Trials 2023; 24: 96. 36750867 10.1186/s13063-023-07074-w PMC9904271

[79] Harris R Rosecrans A Zoltick M , et al. Utilizing telemedicine during COVID-19 pandemic for a low-threshold, street-based buprenorphine program. Drug Alcohol Depend 2022; 230: 109187. DOI: 10.1016/j.drugalcdep.2021.109187. PMC8619879 34890927

[80] Rosecrans A Harris R Saxton RE , et al. Mobile low-threshold buprenorphine integrated with infectious disease services. J Subst Abuse Treat 2022; 133: 108553. 34238629 10.1016/j.jsat.2021.108553 PMC8702567

[81] Tookes HE Bartholomew TS Suarez E , et al. Acceptability, feasibility, and pilot results of the tele-harm reduction intervention for rapid initiation of antiretrovirals among people who inject drugs. Drug Alcohol Depend 2021; 229: 109124. 34781096 10.1016/j.drugalcdep.2021.109124 PMC9102418

[82] Tringale R Subica AM . COVID-19 innovations in medication for addiction treatment at a Skid Row syringe exchange. J Subst Abuse Treat 2021; 121: 108181. 33129635 10.1016/j.jsat.2020.108181 PMC7577224

[83] Belcher AM Coble K Cole TO , et al. Buprenorphine induction in a rural Maryland detention center during COVID-19: Implementation and preliminary outcomes of a Novel Telemedicine treatment program for incarcerated individuals with opioid use disorder. Front Psychiatry 2021; 12: 703685. DOI: 10.3389/fpsyt.2021.703685. PMC8585441 34777036

[84] Yeo EJ Kralles H Sternberg D , et al. Implementing a low-threshold audio-only telehealth model for medication-assisted treatment of opioid use disorder at a community-based non-profit organization in Washington, D.C. Harm Reduct J 2021; 18: 127. 34886850 10.1186/s12954-021-00578-1 PMC8655329

[85] Komaromy M Tomanovich M Taylor JL , et al. Adaptation of a system of treatment for substance use disorders during the COVID-19 pandemic. J Addict Med 2021; 15: 448–451. 33298750 10.1097/ADM.0000000000000791 PMC8562917

[86] Flavin L Tofighi BN Krawczyk NN , et al. Low threshold telemedicine-based opioid treatment for criminal justice involved adults during the COVID-19 pandemic: A case report. J Addict Med 2022; 16: e59–e61. 10.1097/ADM.0000000000000836 PMC8815634 35120069

[87] Watson DP Swartz JA Robison-Taylor L , et al. Syringe service program-based telemedicine linkage to opioid use disorder treatment: Protocol for the STAMINA randomized control trial. BMC Public Health 2021; 21: 630. 33789642 10.1186/s12889-021-10669-0 PMC8010496

[88] Bartholow LAM Huffman RT . The necessity of a trauma-informed paradigm in substance use disorder services. J Am Psychiatr Nurses Assoc 2021: 10783903211036496. 10.1177/10783903211036496 34334012

[89] Lambdin BH Kan D Kral AH . Improving equity and access to buprenorphine treatment through telemedicine at syringe services programs. Subst Abuse Treat Prev Policy 2022; 17: 51. 35841036 10.1186/s13011-022-00483-1 PMC9283820

[90] Levander XA Wheelock H Pope J , et al. Low-threshold buprenorphine via community partnerships and telemedicine-case reports of expanding access to addiction treatment during COVID-19. J Addict Med 2022; 16: e56–e58. 10.1097/ADM.0000000000000811 PMC8815644 34374502

[91] Holmes H Burgess G . Digital exclusion and poverty in the UK: How structural inequality shapes experiences of getting online. Digital Geography and Society 2022; 3: 100041.

[92] Dunham K Giardina M Kolod B , et al. Transitioning clinical care for people who use drugs to telemedicine: Lessons learned one year into the COVID-19 pandemic. Telemed e-Health 2021; 27: 929–933. 10.1089/tmj.2021.0130 34030466

[93] Patton EW Saia K Stein MD . Integrated substance use and prenatal care delivery in the era of COVID-19. J Subst Abuse Treat 2021; 124: 108273. DOI: 10.1016/j.jsat.2020.108273. PMC7979279 33771277

[94] Lopez AM Thomann M Dhatt Z , et al. Understanding racial inequities in the implementation of harm reduction initiatives. Am J Public Health 2022; 112: S173–S181. 10.2105/AJPH.2022.306767 PMC8965181 35349311

[95] Acharya A Izquierdo AM Gonçalves SF , et al. Exploring county-level spatio-temporal patterns in opioid overdose related emergency department visits. *PLOS ONE* 17: e0269509. DOI: 10.1371/journal.pone.0269509. PMC9803238 36584000

[96] Korthuis PT McCarty D Weimer M , et al. Primary care-based models for the treatment of opioid use disorder: A scoping review. Ann Intern Med 2017; 166: 268. 27919103 10.7326/M16-2149 PMC5504692

[97] Goldsamt LA Rosenblum A Appel P , et al. The impact of COVID-19 on opioid treatment programs in the United States. Drug Alcohol Depend 2021; 228: 109049. DOI: 10.1016/j.drugalcdep.2021.109049. PMC8461004 34600258

[98] Bono MH Treitler P Saloner B , et al. Returning home during the pandemic: A thematic analysis describing experiences of people with substance use disorders released early from New Jersey prisons during COVID-19. Health Justice 2023; 11: 11. 36847934 10.1186/s40352-023-00208-x PMC9969013

[99] Eibl JK Morin K Leinonen E , et al. The state of opioid agonist therapy in Canada 20 years after federal oversight. Can J Psychiatry / La Revue Canadienne de Psychiatrie 2017; 62: 444–450. 10.1177/0706743717711167 PMC5528991 28525291

[100] O’Gurek DT . Designing and evaluating COVID-19 protocols for an office-based opioid treatment program in an urban underserved setting. J Am Board Fam Med: JABFM 2021; 34: S136–S140. 10.3122/jabfm.2021.S1.200207 33622828

[101] Hser Y-I Ober AJ Dopp AR , et al. Is telemedicine the answer to rural expansion of medication treatment for opioid use disorder? Early experiences in the feasibility study phase of a National Drug Abuse Treatment Clinical Trials Network trial. Addict Sci Clin Pract 2021; 16: 24. DOI: 10.1186/s13722-021-00233-x. PMC8056373 33879260

[102] Weintraub E Greenblatt AD Chang J , et al. Expanding access to buprenorphine treatment in rural areas with the use of telemedicine. Am J Addict 2018; 27: 612–617. 30265425 10.1111/ajad.12805

[103] Weintraub E Greenblatt AD Chang J , et al. Outcomes for patients receiving telemedicine-delivered medication-based treatment for opioid use disorder: A retrospective chart review. Heroin Addict Relat Clin Probl 2021; 23: 5–12. 33551692 PMC7861202

[104] Aronowitz SV Engel-Rebitzer E Lowenstein M , et al. “We have to be uncomfortable and creative”: Reflections on the impacts of the COVID-19 pandemic on overdose prevention, harm reduction & homelessness advocacy in Philadelphia. SSM Qual Res Health 2021; 1: 100013. 34870265 10.1016/j.ssmqr.2021.100013 PMC8485140

[105] Beieler AM Klein JW Bhatraju E , et al. Evaluation of bundled interventions for patients with opioid use disorder experiencing homelessness receiving extended antibiotics for severe infection. Open Forum Infect Dis 2021; 8: ofab285. 10.1093/ofid/ofab285 PMC8231362 34189180

[106] Mehrotra A Wang B Snyder G . Telemedicine: What should the post- pandemic regulatory and payment landscape look like? Issue Brief. The Commonwealth Fund. 2020 Aug 5. Available at: https://www.senate.mn/commiZees/2021-2022/3095CommiZee_on_Health_and_Human_Services_Finance_and_Policy/Commonwealth%20Fund_Telemedicine.pdf

[107] Wunsch C Wightman R Pratty C , et al. Thirty-day treatment continuation after audio-only buprenorphine telehealth initiation. J Addict Med 2023; 17: 206. 36102540 10.1097/ADM.0000000000001077

[108] Shulman M Wai JM Nunes EV . Buprenorphine treatment for opioid use disorder: An overview. CNS Drugs 2019; 33: 567–580. 31062259 10.1007/s40263-019-00637-z PMC6585403

[109] Kesten JM Holland A Linton M-J , et al. Living Under Coronavirus and Injecting Drugs in Bristol (LUCID-B): A qualitative study of experiences of COVID-19 among people who inject drugs. International Journal of Drug Policy 2021; 98: 103391. 34343945 10.1016/j.drugpo.2021.103391 PMC8289673

[110] Aldabergenov D Reynolds L Scott J , et al. Methadone and buprenorphine-related deaths among people prescribed and not prescribed opioid agonist therapy during the COVID-19 pandemic in England. Int J Drug Policy 2022; 110: 103877. 36265326 10.1016/j.drugpo.2022.103877 PMC9531664

