# Supplementary material for: Understanding the use of telemedicine across different opioid use disorder treatment models: A scoping review
Source: J Telemed Telecare. 2023 Sep 4;31(4):500–14. doi: 10.1177/1357633X231195607 (PMC12044217; doi:10.1177/1357633X231195607)
Supplement: sj-pdf-1-jtt-10.1177_1357633X231195607 - Supplemental material for Understanding the use of telemedicine across different opioid use disorder treatment models: A scoping review [file sj-pdf-1-jtt-10.1177_1357633X231195607.pdf]

Supplementary material

Search used on OVID Medline from inception (1946) to 06/04/23.

1. (narcotic* or opiate* or opioid* or heroin or morphine).mp. [mp=title, book title, abstract, original title, name of substance word, subject heading word, floating sub-heading word, keyword heading word, organism supplementary concept word, protocol supplementary concept word, rare disease supplementary concept word, unique identifier, synonyms]

2. (misuse or abus* or addict* or habit* or withdraw*).mp. [mp=title, book title, abstract, original title, name of substance word, subject heading word, floating sub-heading word, keyword heading word, organism supplementary concept word, protocol supplementary concept word, rare disease supplementary concept word, unique identifier, synonyms]

3. Opioid-Related Disorders/

4. 1 or 2 or 3

5. Buprenorphine/ or Narcotic Antagonists/ or Opiate Substitution Treatment/ or medication assisted treatment.mp.

6. Methadone/

7. 5 or 6

8. 4 and 7

9. Telemedicine/ or Remote Consultation/ or Telehealth*.mp.

10. Videoconferencing/ or telepsychiatry*.mp.

11. 9 or 10

12. 8 and 11

13. limit 12 to (abstracts and English language and humans)

Supplementary Table 1. Details of the included studies.

Reference numbered as appearing in text	Reference	Author	Year	Country	Type	COVID-19 adaptation	Contribution	Model	Name of service
75	1	Aronowitz et al.	2021	USA	Qualitative interviews	Y	Staff at a Philadelphia based low barrier, harm reduction oriented, opioid use disorder (OUD) treatment service identified that an expansion of TMOUD must account for technological barriers and clinician and service attitudes towards providing virtual care to patients regarded as unstable (i.e.,: unhoused, unemployed, or living with additional mental health problems). Failing to do so may increase barriers and inequality access to treatment to the most marginalised.	Low threshold, harm reduction oriented service. Patients often regarded as unstable, high risk, or requiring high intensity interventions.	Prevention Point Philadelphia
44	2	Avalone et al.	2022	USA	Retrospective data analysis	Y	New York City Health + Hospitals (H+ H) is the largest public health system in the United States, and provides substance use disorder services at 10 medically supervised outpatient programs (9 hospital-based and 1 freestanding)<br>and 4 hospital-based opioid treatment programs (OTPs).<br>This article describes the visit attendance rates of multiple SUD outpatient treatment clinics in this system that transitioned from in person to televisits for the duration of the stay at home order during the height of the COVID 19 pandemic.	National/ systems wide service	New York City Health + Hospitals (H+ H) opioid treatment programs
83	3	Belcher et al.	2021	USA	Service innovation and evaluation	Y	Describes the successful implementation of TMOUD in a rural detention center, with treatment engagement and initiation occurring prior to the high-risk period of	Prison based opioid treatment program	The Talbot County Detention Center, Maryland.

							discharge and treatment continuity offered at discharge.		
43	4	Brunet et al.	2020	USA	Service innovation	N	Describes the piloting of tele-buprenorphine programs to veterans with OUD in three rural community based outpatient clinics in the state of Maine. The Veterans Health Affairs (VHA) is an integrated healthcare system with established telemedicine programs which facilitated the implementation of TMOUD.	Hub-spoke TMOUD program<br>Rural community based outpatient clinics.	Department of Veterans Health Affairs (VHA) telemedicine-delivered OUD care
67	5	Cales et al.	2022	USA	Service innovation	Y	Most clinic activities continued as face to face with the exception of a small number of medically vulnerable to COVID-19 people. Obstacles to abstinence included loss of employment, onset of depression, loss of transportation, food insecurity, loss of housing, and difficulty maintaining abstinence.	Systems wide service	NuLease Medical Solutions, Louisville, Kentucky
76	6	Castillo et al.	2020	USA	Service innovation	Y	The clinic provides primary and specialty care to people who inject drugs, including general health screenings, referrals for MOUD, and wound care for skin and soft tissue infections. The pandemic TMOUD clinic filled service gaps while reducing face-to-face clinical interactions and provided students with experience in caring for PWID.	Low threshold, SSP based TMOUD clinic.	The Infectious Disease Elimination Act (IDEA) syringe services program (SSP), Miami, Florida.

51	7	Crowley and Delargy	2020	Ireland	Service innovation	Y	The Irish national system of MOUD is typically initiated by specialist general practitioners, and when appropriate, is handed to community based GPs for ongoing management. Methadone is the first line treatment. The Irish health service developed a hybrid remote model of MOUD provision using telephone triage, urine testing, and electronic prescribing using community pharmacies.	National/ systems wide service	Irish national model of remote care for opioid agonist treatment
56	8	Day et al.	2022	Canada	Service innovation and evaluation	N	Utilizes existing Alberta Health Service telehealth infrastructure within hospitals, community health centres, and clinics across the province to connect a multidisciplinary team with clients requiring MOUD. No in-person component of care. Supports individuals from anywhere in Alberta, with the majority of clients referred from rural and regional home communities. Incorporates a same day start service and a transitional treatment service to minimise delays or breaks in treatment.	A systems wide virtual only opioid dependency program.	Virtual opioid dependency program, Alberta
92	9	Dunham et al.	2021	USA	Service innovation and evaluation	Y	A primary care clinic for people who use drugs using a multidisciplinary team model, providing harm reduction-focused primary care, medications for opioid and alcohol use disorder, and hepatitis C virus (HCV) testing and treatment. This paper describes and evaluates the adaptations made to maintain engagement with and meet the needs of its patients while offering TMOUD.	Harm reduction-focused trauma informed primary care. Low threshold.	Respectful and Equitable Access to Comprehensive Healthcare (REACH) Program, Mount Sinai Hospital, New York City.

52	10	Durand et al.	2022	Ireland	National Delphi study	Y	Stakeholders involved in delivering OUD care in Ireland participated in a Delphi process to decide on whether pandemic related regulatory and policy easing should be retained in the longer term. Consensus was achieved on allowing short initial assessments via telemedicine to diagnose and initiate MOUD, allowing monthly virtual consultations for follow up and the continued use of e-prescribing. Both methadone and buprenorphine would be offered in this way.	National/ systems wide service	Irish national model of remote care for opioid agonist treatment
54	11	Eibl et al.	2017	Canada	Retrospective data analysis	N	Hub-Spoke model offering maintenance MOUD, characterized by frequent clinic visits, observed dosing, urine screening, and limited take-home doses. Telemedicine is practiced with the patient presenting at a secure videoconference site, usually located at a clinic under the supervision of a registered nurse, where they can be connected to a physician in a different location within Ontario, Canada.	National/ systems wide service	-
46	12	Fiacco et al.	2021	USA	Service innovation	Y	The rapid transition to telemedicine of the MOUD service allowed more efficient logistical use of clinical and therapist time such that additional remote services were introduced including hospital e-consultation services, direct connections to primary care, virtual group therapy and virtual individual therapy.	Systems wide service	The Substance Treatment and Recovery (STAR) clinic, South Carolina.
86	13	Flavin et al.	2022	USA	Case report	Y	One of three papers describing different aspects of the New York City Health + Hospitals (H+ H) opioid treatment programs. This case study focusses on low-threshold telemedicine-based opioid treatment with buprenorphine-naloxone, specifically for criminal justice involved adults post-release.	Bridge clinic for criminal justice involved patients	New York City Health + Hospitals (H+ H) opioid treatment programs.

17	14	Gainer et al.	2023	USA	Retrospective data analysis	Y	Treatment programs remained open for in-person care, but with concurrent video or telephone-based (telemedicine) services. Telemedicine was used particularly for patients receiving outpatient, intensive outpatient, and ambulatory withdrawal management services. Urine testing was done in person but could be asynchronous. Patients and practitioners could opt for the mode they preferred. Patients who were homeless, unemployed, or who has higher brief addiction monitor scores were more likely to opt for in-person contact.	Flexible, hybrid model with a shared decision on modality of treatment (virtual or in person)	OneFifteen / Samaritan Behavioral Health, Inc. Dayton, Ohio.
59	15	Ghosh et al.	2021	India	Service innovation	Y	Synchronous, stepwise, mixed model incorporating telephone, video, and face-to-face elements. The provision of MOUD required in person contact.	National/ systems wide service	Drug De-addiction and Treatment Centre, Chandigarh.
68	16	Guille et al.	2020	USA	Non-randomised controlled trial	N	Virtually integrated OUD care into obstetric practices and perinatal care. Outcomes were equivalent for TMOUD compared against treatment as usual.	Hospital based in-reach Multi-disciplinary Perinatal care	**Treatment of Opioid Use Disorder in Pregnant Women via Telemedicine, Charleston, Medical University** of **South Carolina**.
79	17	Harris et al.	2022	USA	Service innovation	Y	The Spot's transitioned patients from a street medicine program to telemedicine during the COVID-19 pandemic. Increased flexibility of service delivery, extended prescription length, and decreased drug testing contributed to high retention rates.	Low-threshold, street-based buprenorphine program.	**Healthcare on the Spot (The Spot), Boston, Massachusetts**
101	18	Hser et al.	2021	USA	Feasibility study	N	A feasibility study to develop and test implementation procedures of TMOUD in preparation for a large-scale, randomised controlled trial in rural primary care. Multiple implementation barriers, include a low detection of opioid use disorder at screening, low rates of TMOUD referral, technology, workflow and capacity, and insurance-coverage issues.	National/ systems wide service	Rural MOUD Telemedicine in Primary Care Phase 1 (Feasibility): NCT04418453

48	19	Kaur et al.	2022	USA	Retrospective data analysis	Y	This study identified a high retention rate in treatment via TMOUD offered through a rural Federally Qualified Health Center in south-central Pennsylvania servicing 56,000 patients, more than 60% patients from rural counties.	National/ systems wide service	Keystone Health, Pennsylvania
45	20	Kennedy et al.	2023	USA	Service innovation	Y	Los Angeles County Department of Health Services established a low-barrier telephone service for all affiliated care providers. First service to provide community health workers entering encampments and linking patients with providers telephonically. Like prior studies, we noted disparities in access to care among racial/ethnic minorities.11–13 Even with this low-threshold approach disparities in accessing MOUD among Black and Latino populations remained.	Systems wide service.<br>Low threshold<br>Community outreach	Los Angeles County Department of Health Services
85	21	Komaromy et al.	2021	USA	Service innovation	Y	Programs across the care continuum for people with substance use disorders which had to innovate quickly during the COVID-19 outbreak to maintain access to care.<br>Programs switched to telehealth with high levels of acceptability and patient retention. Some programs maintained face-to-face visits to see patients with complex problems and to provide injectable medications. A 24-hour hotline supported access to treatment for those released from incarceration early due to the pandemic.<br>Programs included the Faster Paths ‘‘bridge’’ clinic with entry points from street outreach harm-reduction/outreach specialists, the emergency department or through the addiction consult service, the Recovery, Empowerment, Social Services, Prenatal care, Education, Community and Treatment (RESPECT) clinic.	Systems wide service	The Grayken Center for Addiction at the Boston Medical Center.<br>Faster Paths ‘‘bridge’’ clinic.<br>The Recovery, Empowerment, Social Services, Prenatal care, Education, Community and Treatment (RESPECT) clinic.

55	22	LaBelle et al.	2018	Canada	Retrospective data analysis	N	Telemedicine can be effective in providing TMOUD to patients with opioid use disorder and co-occurring mental health disorders.	Systems wide service	Ontario Telemedicine Network
89	23	Lambdin et al.	2022	USA	Retrospective data analysis	Y	A substantial number of syringe service programs implemented TMOUD with buprenorphine after the introduction of a waiver of the in-person exam requirement before treatment induction. TMOUD implementation within SSPs was more likely among services with higher annual budgets and not necessarily associated with need as measured by opioid overdose mortality rates. This indicates that permanent adoption of this waiver, while critical, is insufficient to ensure TMOUD continues within SSPs in the absence of continued and rage fenced funding.	National/systems wide service. Low threshold, community based, harm reduction, culturally appropriate.	SSP based tele-buprenorphine
90	24	Levander et al.	2022	USA	Case report	Y	This study discusses the successful engagement of two patients through community outreach partners (SSP and harm reduction) and TMOUD delivering low threshold buprenorphine.	Low threshold, community based, street outreach workers.	The Harm Reduction and BRidges to Care (HRBR) Clinic, Portland, Oregon.
42	25	Mattocks et al.	2022	USA	Semi-structured interviews	Y	The VHA are well equipped to transition to telemedicine due to pre-existing robust infrastructure. Implementation of TMOUD required an attitudinal and treatment philosophy change towards less restrictive harm reduction approaches. Providers reflected on the need for stringent requirements (e.g., in-person visits, toxicology screens) versus shared decision making with patients.	National/ systems wide service. Hub-spoke	Department of Veterans Health Affairs (VHA) telemedicine-delivered OUD care, USA.

58	26	Mayet et al.	2023	UK	Feasibility study	N	This study took place in a large semi-rural community addictions service (2500km2). Patients were already prescribed opioid substitution treatment and attended an outreach clinic to see a case worker who undertook drug testing and facilitated the telemedicine consult via their laptop. There were no adverse outcomes or differences in clinical outcomes, although connection issues interfered.	Systems wide service. Hub-spoke model. Outreach clinics.	**Telemedicine in Addictions service, East Riding Partnership Community Drug and Alcohol Service, England, UK.**
57	27	Mayet et al.	2021	UK	Qualitative interviews	N	The service was overwhelmingly highly recommended by patients because of convenience and supportive staff.	Systems wide service. Hub-spoke model. Outreach clinics.	**Telemedicine in Addictions service, East Riding Partnership Community Drug and Alcohol Service, England, UK.**
53	28	McDonald et al.	2023	Norway	Service innovation	Y	The use of telemedicine was appreciated due to a reduction in travel time, and opportunities to collaborate with other services. It was less preferred for less stable or more complex patients. Offering patients choice between modalities was seen as important to avoid the social isolation of more vulnerable patients. TMOUD should not prevent offering depot buprenorphine options which were increasingly preferred by some patients during the pandemic.	National/ systems wide service. Norway	**Norwegian national OUD treatment services.**
100	29	O'Gurek	2021	USA	Protocol design and evaluation	Y	The development of pre-arranged, organised workflows in response to emergencies, such as pandemics, supports the crucial service continuation of TMOUD. Incorporating telemedicine, in the context of relaxed federal regulations, has improved addiction care. The protocol implementation was evaluated with retrospective chart review.	OBOT Undeserved urban population.	The Temple Recovery Using Scientific Treatments (TRUST) Clinic, Philadelphia

93	30	Patton et al.	2021	USA	Service innovation	Y	This is a patient-centered integrated SUD and prenatal care service which shifted to a hybrid telemedicine/in-person prenatal care model. Many visits used telemedicine with in-person visits reserved for critical obstetric milestones. Increased take home MOUD was also permitted. The modified service did not cause problems anticipated based on patient stability.	Hospital based in-reach<br>Multi-disciplinary<br>Hybrid	The Recovery, Empowerment, Social Services, Prenatal care, Education, Community and Treatment (RESPECT) clinic, Boston, Massachusetts.
47	31	Poulsen et al.	2023	USA	Service innovation	Y	This paper provides a description of five different Pennsylvanian health providers which developed TMOUD programs in response to COVID-19. Differences in TMOUD protocols, patient eligibility and operationalization of remote drug testing are described. A greater proportion of female, White, and non-Hispanic patients accessed TMOUD.	Systems wide service.	Geisinger integrated health system,<br>Tower Health,<br>Wright Center for Community Health, Lehigh Valley Health Network,<br>WellSpan Health.<br>Pennsylvania.
49	32	Rollston et al.	2022	USA	Service innovation	N	This paper describes a virtual only TMOUD service operating in 23 U.S states. The service provides patient-centred care, including chronic disease management and behavioural health, creation of tech-enabled systems, data-driven decision-making technology and platforms to support patients' needs and treatment goals.	National/ systems wide service.<br>Hub-spoke model.<br>Fully virtual service.	Bicycle health
77	33	Suarez, E. et al.	2023	USA	Retrospective data analysis	N	Tele harm reduction intervention to provide buprenorphine in a non-stigmatising environment and promote HIV viral suppression among people who inject drugs (PWID) accessing a syringe services program (SSP). This pilot suggests that an SSP may be an acceptable and feasible venue for tele harm reductio to increase uptake of buprenorphine by PWID and promote retention in care.	A multicomponent, telehealth-based, peer-driven intervention offered through a syringe services program (SSP)	Infectious Disease Elimination Act (IDEA) Miami Syringe Services Program

62	34	Tofighi et al.	2022	USA	Service evaluation	Y	Comprehensive low threshold TMOUD service including opioid overdose education, home induction of buprenorphine, naloxone administration and supply, primary and specialty care (eg, psychiatry, HIV, and pain management) and a bridge into community services for underserved people (eg, unstably housed, justice-involved, Latinx, and African American).<br>Critically, bureaucratic hurdles, waiting on the telephone, accessing transport, or accessing a pharmacy all interfere with the success of TMOUD, yet are not directly addressed by initiatives to address the digital divide.	Systems wide service.<br>Integrated primary and secondary care<br>Low threshold service<br>Bridge into community services	NYC Public Hospital System
61	35	Tofighi et al.	2022	USA	Service evaluation	Y	A buprenorphine bridging service providing same day prescribing for patients who found out about the service through word of mouth, correctional health or reentry services, community organizations supporting reentry, online searches, homeless shelter staff or social service and harm reduction programs. Patients move on from this service to community MOUD programs.	Systems wide service.<br>Low-Threshold Tele-Buprenorphine Bridge Clinic	NYC Public Hospital System
78	36	Tookes et al.	2023	USA	Feasibility study	N	This study is intended to increase availability and accessibility of ART, MOUD, and HCV cure for PWID with HIV using trusted SSPs. The overall goal is to compare the efficacy of the THR intervention in achieving HIV viral suppression among PWID with uncontrolled HIV infection in accessing services at an SSP compared to current standard of care (i.e., off-site linkage to HIV care).	Low threshold, comprehensive treatment/ harm reduction model for PWID with HIV in trusted syringe service programs.	T-SHARP: IDEA Miami, IDEA Tampa the SPOT located in Ft. Lauderdale, Florida.

81	37	Tookes et al.	2021	USA	RCT study protocol	N	Participant feedback during this pilot allowed a refinement of the tele-harm reduction intervention including adding phlebotomy , outreach staff with iPads, WiFi hotspots, and disposable headphones, compassionate delivery of care enhanced via mobile SSP, partnerships with safety-net hospitals, substance use disorder treatment programs and shelters.	tele-harm reduction intervention for rapid initiation of antiretrovirals among people who inject drugs	T-SHARP pilot:Test and Treat site at the IDEA Miami SSP in partnership with the Florida Department of Health.
82	38	Tringale and Subica	2021	USA	Service innovation	Y	Los Angeles' Skid Row Syringe exchange patients were able to access MOUD through an innovative telephone booth model facilitating TMOUD.<br>A novel coordinated pharmacy dispensing model was effective with TMOUD patients.	Low threshold, community based, street outreach, syringe service program.	**Centre for Harm Reduction, Homeless Health Care Los Angeles (HHCLA)**
87	39	Watson et al.	2021	USA	RCT study protocol	N	This study intends to shorten the time between someone deciding to access treatment in a syringe programme and MOUD induction via telemedicine. The study also provided evidence regarding the positive and negative effects of pandemic-related regulatory easing.	SSP, linkage to TMOUD for buprenorphine and facilitation of in person assessment at an OTP for methadone.	STAMINA study at three SSP sites in Chicago, Illinois.
102	40	Weintraub et al.	2018	USA	Retrospective data analysis	N	This study evaluated a program that began providing buprenorphine treatment to patients at a drug treatment center in rural Maryland via telemedicine in August 2015. Findings suggest that treatment with buprenorphine can be effectively delivered by telemedicine to patients with opioid use disorders in a rural drug treatment program.	Hub-spoke model facilitating rural provision or TMOUD	Wells House Intensive Outpatient Treatment Program, Maryland
103	41	Weintraub et al.	2021	USA	Retrospective data analysis	N	This study provides further evidence of the effectiveness of TMOUD offered via a hub-spoke model that are comparable to face-to-face treatment, providing further support for this model in improving access to underserved rural areas,	Hub-spoke model facilitating rural provision or TMOUD	Wells House Intensive Outpatient Treatment Program, Maryland

60	42	Wightman et al.	2021	USA	Service innovation	Y	This is a retrospective cohort study of an ED-callback pilot project providing real-time telehealth delivered buprenorphine initiation and referral to community harm reduction and addiction treatment services for ED patients treated for an opioid overdose	Emergency department linkage to treatment via telemedicine	Emergency department callback pilot project, Rhode Island.
50	43	Williams et al.	2023	USA	Retrospective data analysis	N	A virtual-first TMOUD platform. Medical visits, urine drug screens, clinical governance, electronic health records and care coordination have been designed for virtual care. Unsuitable for PWOUD requiring a higher level of care. Buprenorphine offered, seen weekly during stabilization and then stepped down to monthly visits under a nurse care manager model.	TMOUD by design.	Ophelia
84	44	Yeo et al.	2021	USA	Service innovation	Yes	Flexible, low-threshold model of audio-only telehealth care to increase access to MOUD. Most staff share lived experiences with clients, including recovery from substance use, and from similar communities. High levels of low socioeconomic status, homelessness, previous incarceration, and chronic health conditions. Majority black and male patients.	Low threshold, community based, harm reduction, lived experience. Telephone only.	Honoring Individual Power & Strength<br>(HIPS) clinic, Washington, D.C.
71	45	Zheng et al.	2017	USA	Retrospective data analysis	No	Standard treatment program incorporating urine drug screenings through local community mental health facilities in a rural setting except that the psychiatrist delivers addiction assessment and care in a group setting through videoconferencing. No significant difference in terms of additional substance use, and retention in treatment whether MOUD was delivered in person or virtually.	Hub-spoke model. Clinician remotely providing virtual groups consultations for MOUD.	Comprehensive Opioid Addiction Treatment (COAT) Program, West Virgina.

References

1. Aronowitz SV, Engel-Rebitzer E, Dolan A, et al. Telehealth for opioid use disorder treatment in low-barrier clinic settings: an exploration of clinician and staff perspectives. *Harm reduction journal* 2021; 18: 119.

2. Avalone L, King C, Popeo D, et al. Increased Attendance During Rapid Implementation of Telehealth for Substance Use Disorders During COVID-19 at the Largest Public Hospital System in the United States. *Substance use & misuse* 2022; 57: 1322–1327.

3. Belcher AM, Coble K, Cole TO, et al. Buprenorphine Induction in a Rural Maryland Detention Center During COVID-19: Implementation and Preliminary Outcomes of a Novel Telemedicine Treatment Program for Incarcerated Individuals With Opioid Use Disorder. *Frontiers in Psychiatry*; 12. Epub ahead of print 2021. DOI: 10.3389/fpsyt.2021.703685.

4. Brunet N, Moore D, Wischik D, et al. Increasing buprenorphine access for veterans with opioid use disorder in rural clinics using telemedicine. *Substance Abuse* 2020; 43: 1–8.

5. Cales RH, Cales SC, Shreffler J, et al. The COVID-19 pandemic and opioid use disorder: Expanding treatment with buprenorphine, and combining safety precautions with telehealth. *Journal of Substance Abuse Treatment*; 133. Epub ahead of print 2022. DOI: 10.1016/j.jsat.2021.108543.

6. Castillo M, Conte B, Hinkes S, et al. Implementation of a medical student-run telemedicine program for medications for opioid use disorder during the COVID-19 pandemic. *Harm Reduction Journal*; 17. Epub ahead of print 2020. DOI: 10.1186/s12954-020-00438-4.

7. Crowley D, Delargy I. A national model of remote care for assessing and providing opioid agonist treatment during the COVID-19 pandemic: A report. *Harm Reduction Journal*; 17. Epub ahead of print 17 July 2020. DOI: 10.1186/s12954-020-00394-z.

8. Day N, Wass M, Smith K. Virtual opioid agonist treatment: Alberta's virtual opioid dependency program and outcomes. *Addiction science & clinical practice* 2022; 17: 40.

9. Dunham K, Giardina M, Kolod B, et al. Transitioning Clinical Care for People Who Use Drugs to Telemedicine: Lessons Learned One Year into the COVID-19 Pandemic. *Telemedicine and e-Health* 2021; 27: 929–933.

10. Durand L, Keenan E, Boland F, et al. Consensus recommendations for opioid agonist treatment following the introduction of emergency clinical guidelines in Ireland during the COVID-19 pandemic: A national Delphi study. *International Journal of Drug Policy* 2022; 106: 103768.

11. Eibl JK, Gauthier G, Pellegrini D, et al. The effectiveness of telemedicine-delivered opioid agonist therapy in a supervised clinical setting. *Drug and Alcohol Dependence* 2017; 176: 133–138.

12. Fiacco L, Pearson BL, Jordan R. Telemedicine works for treating substance use disorder: The STAR clinic experience during COVID-19. *Journal of Substance Abuse Treatment* 2021; 125: 108312.

13. Flavin L, Tofighi BN, Krawczyk NN, et al. Low Threshold Telemedicine-based Opioid Treatment for Criminal Justice Involved Adults During the COVID-19 Pandemic: A Case Report. *Journal of Addiction Medicine* 2022; 16: e59–e61.

14. Gainer DM, Wong C, Embree JA, et al. Effects of Telehealth on Dropout and Retention in Care among Treatment-Seeking Individuals with Substance Use Disorder: A Retrospective Cohort Study. *Substance Use & Misuse* 2023; 1–10.

15. Ghosh A, Mahintamani T, B.N. S, et al. Telemedicine-assisted stepwise approach of service delivery for substance use disorders in India. *Asian Journal of Psychiatry* 2021; 58: 102582.

16. Guille C, Simpson AN, Douglas E, et al. Treatment of Opioid Use Disorder in Pregnant Women via Telemedicine: A Nonrandomized Controlled Trial. *JAMA Network Open* 2020; 3: e1920177–e1920177.

17. Harris R, Rosecrans A, Zoltick M, et al. Utilizing telemedicine during COVID-19 pandemic for a low-threshold, street-based buprenorphine program. *Drug and Alcohol Dependence*; 230. Epub ahead of print 1 January 2022. DOI: 10.1016/j.drugalcdep.2021.109187.

18. Hser Y-I, Ober AJ, Dopp AR, et al. Is telemedicine the answer to rural expansion of medication treatment for opioid use disorder? Early experiences in the feasibility study phase of a National Drug Abuse Treatment Clinical Trials Network trial. *Addiction Science & Clinical Practice*; 16. Epub ahead of print 20 April 2021. DOI: 10.1186/s13722-021-00233-x.

19. Kaur J, Mania I, Tirupathi R, et al. Impact of telemedicine on retention in Medications for Opioid Use Disorder (MOUD) treatment with buprenorphine in the times of COVID-19 pandemic: A retrospective chart review. *Journal of Rural Mental Health* 2022; 46: 75–81.

20. Kennedy AJ, George JS, Rossetti G, et al. Providing Low-barrier Addiction Treatment Via a Telemedicine Consultation Service During the COVID-19 Pandemic in Los Angeles, County: An Assessment 1 Year Later. *Journal of Addiction Medicine* 2023; 17: e64.

21. Komaromy M, Tomanovich M, Taylor JL, et al. Adaptation of a System of Treatment for Substance Use Disorders During the COVID-19 Pandemic. *Journal of addiction medicine* 2021; 15: 448–451.

22. LaBelle B, Franklyn AM, PKH Nguyen V, et al. Characterizing the Use of Telepsychiatry for Patients with Opioid Use Disorder and Cooccurring Mental Health Disorders in Ontario, Canada. *International Journal of Telemedicine and Applications* 2018; 2018: e7937610.

23. Lambdin BH, Kan D, Kral AH. Improving equity and access to buprenorphine treatment through telemedicine at syringe services programs. *Substance abuse treatment, prevention, and policy* 2022; 17: 51.

24. Levander XA, Wheelock H, Pope J, et al. Low-Threshold Buprenorphine via Community Partnerships and Telemedicine-Case Reports of Expanding Access to Addiction Treatment During COVID-19. *Journal of Addiction Medicine* 2022; 16: e56–e58.

25. Mattocks KM, Moore DT, Wischik DL, et al. Understanding opportunities and challenges with telemedicine-delivered buprenorphine during the COVID-19 pandemic. *Journal of substance abuse treatment* 2022; 139: 108777.

26. Mayet S, Gledhill A, McCaw I, et al. Telemedicine in addictions: Feasibility randomised controlled trial. *Heroin Addiction and Related Clinical Problems*.

27. Mayet S, Mccaw I, Hashmani Z, et al. Patient experience of telemedicine in addictions. *BJPsych open* 2021; 7: S269–S270.

28. McDonald R, Bech AB, Clausen T. *Flexible delivery of opioid agonist treatment during COVID-19 in Norway: A cross-sectional survey of provider experiences*. Preprint, In Review. Epub ahead of print 18 January 2023. DOI: 10.21203/rs.3.rs-2212348/v1.

29. O'Gurek DT. Designing and Evaluating COVID-19 Protocols for an Office-Based Opioid Treatment Program in an Urban Underserved Setting. *Journal of the American Board of Family Medicine : JABFM* 2021; 34: S136–S140.

30. Patton EW, Saia K, Stein MD. Integrated substance use and prenatal care delivery in the era of COVID-19. *Journal of Substance Abuse Treatment*; 124. Epub ahead of print 1 May 2021. DOI: 10.1016/j.jsat.2020.108273.

31. Poulsen MN, Santoro W, Scotti R, et al. Implementation of Telemedicine Delivery of Medications for Opioid Use Disorder in Pennsylvania Treatment Programs During COVID-19. *J Addict Med* 2023; 17: e110–e118.

32. Rollston R, Gallogly W, Hoffman L, et al. Collaborative, patient-centred care model that provides tech-enabled treatment of opioid use disorder via telehealth. *BMJ Innovations*; 8. Epub ahead of print 1 April 2022. DOI: 10.1136/bmjinnov-2021-000816.

33. Suarez E, Bartholomew TS, Plesons M, et al. Adaptation of the Tele-Harm Reduction intervention to promote initiation and retention in buprenorphine treatment among people who inject drugs: a retrospective cohort study. *Annals of Medicine* 2023; 55: 733–743.

34. Tofighi B, McNeely J, Walzer D, et al. A Telemedicine Buprenorphine Clinic to Serve New York City: Initial Evaluation of the NYC Public Hospital System's Initiative to Expand Treatment Access During the COVID-19 Pandemic. *Journal of addiction medicine* 2022; 16: e40–e43.

35. Tofighi B, McNeely J, Yang J, et al. Outcomes of a NYC Public Hospital System Low-Threshold Tele-Buprenorphine Bridge Clinic at 1 Year. *Substance Use & Misuse* 2022; 57: 1337–1340.

36. Tookes HE, Oxner A, Serota DP, et al. Project T-SHARP: study protocol for a multi-site randomized controlled trial of tele-harm reduction for people with HIV who inject drugs. *Trials* 2023; 24: 96.

37. Tookes HE, Bartholomew TS, Suarez E, et al. Acceptability, feasibility, and pilot results of the tele-harm reduction intervention for rapid initiation of antiretrovirals among people who inject drugs. *Drug and Alcohol Dependence* 2021; 229: 109124.

38. Tringale R, Subica AM. COVID-19 innovations in medication for addiction treatment at a Skid Row syringe exchange. *Journal of substance abuse treatment* 2021; 121: 108181.

39. Watson DP, Swartz JA, Robison-Taylor L, et al. Syringe service program-based telemedicine linkage to opioid use disorder treatment: protocol for the STAMINA randomized control trial. *BMC public health* 2021; 21: 630.

40. Weintraub E, Greenblatt AD, Chang J, et al. Expanding access to buprenorphine treatment in rural areas with the use of telemedicine. *American Journal on Addictions* 2018; 27: 612–617.

41. Weintraub E, Greenblatt AD, Chang J, et al. Outcomes for patients receiving telemedicine-delivered medication-based treatment for Opioid Use Disorder: A retrospective chart review. *Heroin Addict Relat Clin Probl* 2021; 23: 5–12.

42. Wightman RS, Jacka B, Uber J, et al. Tele-buprenorphine for emergency department overdose visit follow up and treatment initiation. *American Journal of Emergency Medicine* 2021; 50: 409–412.

43. Williams AR, Aronowitz S, Gallagher R, et al. A Virtual-First Telehealth Treatment Model for Opioid Use Disorder. *J GEN INTERN MED* 2023; 38: 814–816.

44. Yeo EJ, Kralles H, Sternberg D, et al. Implementing a low-threshold audio-only telehealth model for medication-assisted treatment of opioid use disorder at a community-based non-profit organization in Washington, D.C. *Harm reduction journal* 2021; 18: 127.

45. Zheng W, Nickasch M, Lander L, et al. Treatment Outcome Comparison Between Telepsychiatry and Face-to-face Buprenorphine Medication-assisted Treatment for Opioid Use Disorder: A 2-Year Retrospective Data Analysis. *Journal of Addiction Medicine* 2017; 11: 138–144.

Supplementary Table 2. Service characteristics syringe service programs/ harm reduction/ street-based services

Service characteristics	Prevention Point Philadelphia	Faster Paths bridge clinic. Boston. Massachusetts	The Infectious Disease Elimination Act (IDEA) syringe services	T-SHARP research project: IDEA Miami, IDEA Tampa the	Centre for Harm Reduction, Homeless Health Care Los Angeles	STAMINA study at three SSP sites in Chicago. Illinois.	Honoring Individual Power & Strength (HIPS) clinic.	Healthcare on the Spot (The Spot), Baltimore	The Harm Reduction and BRidges to Care (HRBR) Clinic, Portland.	Los Angeles County Department of Health Services
**References**	Aronowitz et al. 2021	Komaromy et al., 2021	Castillo et al., 2020; Suarez et al., 2023; Tookes et al., 2021	Tookes et al., 2021, 2023	Tringale and Subica, 2021	Watson et al., 2021	Yeo et al. 2021	Harris et al., 2022; Rosecrans et al., 2021	Levander et al., 2022	Kennedy et al., 2023
**Geographic setting**										
**Urban**	Yes	Yes	Yes	Yes	Yes	Yes	Yes	Yes	Yes	Yes
**Rural**										
**Mixed**										
**Threshold of care**										
**Threshold of care: High**										
**Threshold of care: Low**	Yes	Yes	Yes	Yes	Yes	Yes	Yes	Yes	Yes	Yes
**Same-day treatment entry Prescription at first visit**		Yes	Yes	Yes		Yes		Yes		Yes
**Home-induction of available**			Yes	Yes		Yes		Yes		Yes
**Patient's goals prioritised: Non-judgmental attitude**	Yes		Yes	Yes		Yes		Yes		Yes
**Patient's goals prioritised: Reduction in illicit opioid use as acceptable goal**	Yes		Yes	Yes		Yes		Yes		
**Use of other substances does not result in treatment cessation**	Yes		Yes	Yes		Yes		Yes		
**Flexible- Rapid re-initiation of treatment if missed visit**			Yes	Yes				Yes		

**Availability in non-traditional settings** Buprenorphine prescribed from emergency department, syringe exchange program, mobile units, etc.	Yes		Yes	Yes	Yes	Yes	Yes	Yes		Yes
**Enrolment criteria/ philosophy**										
**Inclusion health focus:.**	Yes	Yes	Yes	Yes	Yes	Yes	Yes	Yes		Yes
Service tailors interventions/programs to different groups according to need.	Yes									
**Target population**										
**Target population: People who inject drugs**	Yes		Yes	Yes	Yes	Yes	Yes	Yes		
**Target population: People experiencing homelessness**	Yes		Yes	Yes	Yes			Yes		Yes
**Target population: People with substance use disorder during pregnancy**										
**Target population: People with HIV**	Yes		Yes	Yes				Yes		
**Target population: People with HCV**								Yes		
**Target population: People involved with criminal justice**	Yes							Yes		
**Target population: People involved with sex work**							Yes			
**Service setting**										
**Service setting: Syringe service programs**	Yes		Yes	Yes	Yes	Yes	Yes	Yes	Yes	
**Service setting: Harm reduction services**	Yes	Yes					Yes			
**Service setting: Street-based**	Yes	Yes	Yes	Yes			Yes	Yes	Yes	Yes

**Service setting: Other community settings**		Yes								
**Service setting: Emergency departments**		Yes								
**Service setting: Hospital based (including obstetrics)**		Yes								
**Service setting: Fully virtual**										
**Service setting: National or systems wide**										Yes
**Type(s) of care**										
**Medical case management:**	Yes		Yes	Yes	Yes			Yes		
**Bridge clinic model/ Continuity of care:**		Yes							Yes	
**Crisis stabilisation** (acutely intoxicated/ in withdrawals)										
**Coordinated Care**)	Yes	Yes	Yes	Yes	Yes		Yes	Yes	Yes	
**Partnership working (also integrated care**			Yes	Yes	Yes	Yes		Yes	Yes	Yes
**Multi-disciplinary Care:** 2 disciplines working together (e.g. Psych & GUM)					Yes					
**Telemedicine specific**										
**Telemedicine facilitator**	Yes	Yes	Yes	Yes	Yes	Yes	Yes		Yes	Yes
**Hybrid: All aspects**		Yes	Yes	Yes						
**Hybrid: Non-prescriber only**	Yes				Yes	Yes			Yes	
**Audio-only**							Yes	Yes		Yes
**Audio and video by choice**									Yes	
**Video only**										

**Must have a rationale for telemedicine appointment**										
**Patient may choose telemedicine or in person**		Yes	Yes	Yes						
**Allows new patients (treatment induction)**		Yes	Yes	Yes		Yes		Yes	Yes	Yes
**Toxicology screening process**										
**Clinically indicated only**			Yes	Yes						
**Waived**								Yes		Yes
**Medication offered**										
**MOUD: Buprenorphine s/l**	Yes	Yes	Yes	Yes	Yes	Yes	Yes	Yes	Yes	Yes
**MOUD: Methadone**										
**MOUD: Buprenorphine injectable depot**										
**MOUD: only following in-person visit**										
**Harm reduction interventions**										
**Harm reduction interventions: Naloxone**	Yes	Yes	Yes	Yes	Yes			Yes	Yes	
**Harm reduction interventions: Overdose awareness and response**	Yes	Yes	Yes	Yes	Yes			Yes	Yes	
**Poly-substance use (co-occurring SUD such as amphetamine and cocaine) or alcohol**		Yes	Yes	Yes						
**Harm reduction interventions: sterile injection equipment**	Yes		Yes	Yes	Yes	Yes		Yes	Yes	
**Harm reduction interventions: BBV testing**	Yes	Yes	Yes	Yes				Yes	Yes	
**Harm reduction interventions: HCV cure**	Yes	Yes	Yes	Yes				Yes		

**Harm reduction interventions: ART- HIV suppression/ PrEP**		Yes	Yes	Yes				Yes		
**Harm reduction interventions: Woundcare**	Yes	Yes	Yes	Yes	Yes			Yes		
**Harm reduction interventions: fentanyl strips**										
**Harm reduction interventions: Digital overdose response system**										
**Not described**										Yes
**Meeting other clinical needs**										
**Linking to community MOUD services**		Yes				Yes		Yes		
**Meeting other clinical needs: onsite phlebotomy**			Yes	Yes				Yes		
**Meeting other clinical needs: flexible medication management protocols such as medication lockers**			Yes	Yes						
**Meeting other clinical needs: flexible medication management protocols such as medication delivery (drop off)**			Yes	Yes						
**Meeting other clinical needs: Reproductive healthcare and birth control**	Yes	Yes	Yes	Yes				Yes		
**Primary care needs (including managing co-morbidities)**		Yes	Yes	Yes						
**Mental health needs (onsite or virtual psychologist/psychiatrist)**			Yes	Yes						
**Chronic pain needs**			Yes	Yes						
**Vaccinations (including for COVID-19)**								Yes		
**Meeting non-clinical needs**										

**Meeting non-clinical needs: social worker**			Yes	Yes						
**Meeting non-clinical needs: Psychosocial assessment**										
**Meeting non-clinical needs: Housing services**	Yes		Yes	Yes	Yes					
**Meeting non-clinical needs: Mail services**	Yes									
**Meeting non-clinical needs: Police Assisted Diversion (PAD)**	Yes									
**Meeting non-clinical needs: Food, clothing, hygiene**					Yes					
**Meeting non-clinical needs: Transport**						Yes				
**Meeting non-clinical needs: wifi**					Yes					
**Pet services**										
**Engagement**										
**Outreach**	Yes	Yes	Yes	Yes	Yes			Yes		Yes
**Patient navigators: to coordinate services and guide patients through the complex health care system, thereby removing barriers to accessing quality care and treatment.**										Yes
**Mobile services (harm reduction, primary care, BBV etc)**	Yes		Yes	Yes				Yes		Yes
**Drop in service**	Yes		Yes	Yes				Yes		
**Peer support**	Yes		Yes	Yes			Yes	Yes		
**Shared decision making**	Yes		Yes	Yes						
**Round the clock emergency support**										
**Non-MOUD therapy**										

**Virtual group or individual therapy**							Yes			
**Counselling**			Yes	Yes			Yes			

Supplementary Table 3. Service characteristics: National and systems level services

**Service characteristics**	**The Substance Treatment and**	**Drug De-addiction and Treatment**	**New York City Health + Hospitals H*H SUD**	**Irish national model of remote care**	**Department of Veterans Health Affairs (HA) telemedicin**	**Hub spoke maintenanc e model or care, Ontario.**	**Los Angeles County Department of Health**	**Norwegian national OUD treatment**	**Geisinger integrated health system. Pennsylvani**	**Tower Health. Pennsylvani a.**	**Wright Center for Community Health, Pennsylvani**	**Lehigh Valley Health Network, Pennsylvani**	**WellSpan Health, Pennsylvani a.**	**Ontario Telemedicin e Network**
**References**	Fiacco et al., 2021	Ghosh et al., 2021	Avalone et al., 2022	Crowley and Delargy, 2020 Durand et al., 2022	Mattocks et al., 2022 Brunet 2020,Chan et al., 2022	Eibl et al., 2017	Kennedy et al., 2023	McDonald et al., 2023	Poulsen et al., 2023	Poulsen et al., 2023	Poulsen et al., 2023	Poulsen et al., 2023	Poulsen et al., 2023	LaBelle et al., 2018
**Geographic setting**														
**Urban**			Yes				Yes							
**Rural**					Yes									
**Mixed**	Yes	Yes		Yes		Yes		Yes	Yes	Yes	Yes	Yes	Yes	Yes
**Threshold of care**														
**Threshold of care: High**						Yes								
**Threshold of care: Low**				Yes			Yes							
**Same-day treatment entry Prescription at first visit**				Yes			Yes							
**Home-induction of buprenorphine available**							Yes							
**Patient's goals prioritised: Non-judgmental attitude**				Yes			Yes							
**Patient's goals prioritised:Reduction in illicit opioid use as acceptable goal**				Yes										
**Use of other substances does not result in treatment cessation**														

**Flexible- Rapid re-initiation of treatment if missed visit**														
**Availability in non-traditional settings** Buprenorphine prescribed from emergency department, syringe exchange program, mobile units, etc.							Yes							
**Threshold of care: Unclear**	Yes	Yes			Yes			Yes	Yes	Yes	Yes	Yes	Yes	Yes
**Enrolment criteria/ philosophy**														
**Inclusion health focus:** Trauma informed principles, culturally appropriate, peer support. Often targeting marginalised easily ignored groups and sensitive to social determinants of health including digital divide. Staff may share lived experiences with clients, including recovery from substance use, and come from similar communities as the clients.							Yes							
Service tailors interventions/programs to different groups according to need.		Yes												
Not described				Yes	Yes									
**Target population**														
**Target population: People experiencing homelessness**							Yes							
**Target population: Veterans with OUD**					Yes									
**Service setting**														
**Service setting: Street-based**							Yes							
**Service setting: Other community settings**					Yes									
**Service setting: Emergency departments**									Yes	Yes		Yes	Yes	

**Service setting: Hospital based (including obstetrics)**	Yes		Yes					Yes	Yes	Yes		Yes	Yes	
**Service setting: Primary care**			Yes	Yes	Yes	Yes		Yes	Yes	Yes	Yes	Yes	Yes	
**Service setting: Dedicated treatment facility**				Yes		Yes			Yes	Yes		Yes	Yes	
**Service setting: National or systems wide**	Yes	Yes	Yes	Yes	Yes	Yes	Yes	Yes	Yes	Yes	Yes	Yes	Yes	Yes
**Type(s) of care**														
**Medical case management:**		Yes			Yes	Yes		Yes	Yes		Yes	Yes		
**Coordinated Care:**	Yes	Yes		Yes	Yes	Yes		Yes	Yes		Yes	Yes		
**Partnership working (also integrated care):**	Yes						Yes	Yes		Yes		Yes		
**Multi-disciplinary Care:**	Yes	Yes			Yes				Yes					
**Shared Care:**	Yes			Yes	Yes	Yes		Yes						
**Chronic Care:**				Yes	Yes	Yes		Yes	Yes		Yes			Yes
**Physician centric or service efficiency focussed:**														Yes
**Stepwise direct care model:**		Yes												
**Office-based opioid treatment (OBOT):**	Yes		Yes	Yes	Yes	Yes		Yes	Yes	Yes	Yes	Yes	Yes	Yes
**Opioid Treatment Programs: Intensive outpatient**			Yes											

**Telemedicine specific**														
**Telemedicine facilitator**	Yes				Yes		Yes							
**Hybrid: All aspects**	Yes	Yes		Yes		Yes		Yes	Yes	Yes	Yes	Yes	Yes	
**Hybrid: Non-prescriber only**					Yes									Yes
**Audio-only**							Yes							
**Audio and video by choice**				Yes				Yes	Yes	Yes	Yes	Yes	Yes	
**Video only**					Yes	Yes								
**Must have a rationale for telemedicine appointment**								Yes			Yes			
**Patient may choose telemedicine or in person**	Yes			Yes										
**Allows new patients (treatment induction)**	Yes	Yes		Yes	Yes	Yes	Yes				Yes			
**Allows only patients stabilised on MOUD**									Yes	Yes				
**Allows only patients already with the service**														
**E-consultation to other hospital specialties or primary care**	Yes													
**Toxicology screening process**														
**Clinically indicated only**				Yes	Yes	Yes		Yes	Yes				Yes	
**Contingency management**				Yes		Yes					Yes		Yes	
**Mandatory random testing**										Yes	Yes			
**Asynchronous** for example, an individual could have a remote treatment encounter and present at a different date and time to provide a urine sample												Yes	Yes	
**Novel/ technological support**									Yes					

**Waived**							Yes							
**Medication offered**														
**MOUD: Buprenorphine s/l**		Yes	Yes	Yes	Yes	Yes	Yes	Yes	Yes	Yes	Yes	Yes	Yes	Yes
**MOUD: Methadone**		Yes	Yes	Yes		Yes		Yes						Yes
**MOUD: Buprenorphine injectable depot**								Yes	Yes	Yes	Yes	Yes	Yes	
**MOUD: only following in-person visit**		Yes												
**Harm reduction interventions**														
**Harm reduction interventions: Naloxone**				Yes										
**Harm reduction interventions: Overdose awareness and response**				Yes										
**Not described**	Yes	Yes	Yes		Yes	Yes	Yes	Yes	Yes	Yes	Yes	Yes	Yes	Yes
**Meeting other clinical needs**														
**Primary care needs (including managing co-morbidities)**					Yes				Yes		Yes	Yes		
**Mental health needs (onsite or virtual psychologist/ psychiatrist)**												Yes		Yes
**Chronic pain needs**												Yes		
**Specialty care needs**												Yes		
**Perinatal care**									Yes			Yes		
**Vaccinations (including for COVID-19)**														
**Meeting non-clinical needs**														
**Meeting non-clinical needs: social worker**		Yes						Yes						
**Meeting non-clinical needs: Psychosocial assessment**		Yes												
**Engagement**														

**Outreach**							Yes							
**Patient navigators**							Yes							
**Mobile services (harm reduction, primary care, BBV etc)**							Yes							
**Non-MOUD therapy**														
**Psychosocial/behavioural therapy**	Yes	Yes						Yes						
**Virtual group or individual therapy**	Yes													
**Counselling**			Yes											

Supplementary Table 4. Service characteristics: virtual only

**Service characteristics**	**Virtual opioid dependency program. Alberta**	**Bicycle health**	**Ophelia Health**
**References**	Day et al., 2022	Rollston et al., 2022.	Williams et al., 2023.
**Urban**			
**Rural**			
**Mixed**	Yes	Yes	Yes
**Threshold of care**			
**Threshold of care: High**			
**Threshold of care: Low**	Yes		
**Same-day treatment entry Prescription at first visit**	Yes		
**Home-induction of available**	Yes	Yes	Yes
**Patient's goals prioritised: Non-judgmental attitude**	Yes		
**Patient's goals prioritised:Reduction in illicit opioid use as acceptable goal**	Yes		

**Use of other substances does not result in treatment cessation**	Yes		
**Flexible- Rapid re-initiation of treatment if missed visit**	Yes		
**Availability in non-traditional settings** Buprenorphine prescribed from emergency department, syringe exchange program, mobile units, etc.			
**Threshold of care: Unclear**		Yes	Yes
**Enrolment criteria/ philosophy**			
**Inclusion health focus:**	Yes		
A level of care which qualifies the patient for telehealth: This may include a low acuity patient focus, a limit of patient complexity allowed or a level of technological competence		Yes	Yes
Service tailors interventions/programs to different groups according to need.	Yes		
**Service setting: Fully virtual**	Yes	Yes	Yes
**Service setting: National or systems wide**	Yes	Yes	Yes
**Type(s) of care**			
**Medical case management:**	Yes	Yes	Yes
**Bridge clinic model:**	Yes		
**Coordinated Care:**	Yes	Yes	Yes
**Partnership working (also integrated care):**	Yes	Yes	Yes
**Chronic Care:**		Yes	
**Physician centric or service efficiency focussed:**			Yes
**Office-based opioid treatment (OBOT):**	Yes	Yes	Yes
**Telemedicine specific**			

**Telemedicine facilitator**	Yes		Yes
**Hybrid: All aspects**			
**Hybrid: Non-prescriber only**			
**Audio-only**			
**Audio and video by choice**	Yes		
**Video only**		Yes	Yes
**Allows new patients (treatment induction)**	Yes		Yes
**Registries eg** date of last patient appointment, future appointments, appointment no-shows, employment status, history of anxiety or depression, use of illicit substances, and history of intravenous drug use.		Yes	
**Dedicated smartphone app**		Yes	
**Chat channels to connect with support staff**		Yes	
**Pharmacy finder tool (stock availability, proximity, non-stigmatising service)**		Yes	
**After hours troubleshooting line**		Yes	
**Evidence-based buprenorphine induction tool (mobile app)**		Yes	
**Toxicology screening process**			
**Clinically indicated only**	Yes	Yes	Yes
**Contingency management**			
**Mandatory random testing**		Yes	
**Asynchronous** for example, an individual could have a remote treatment encounter and present at a different date and time to provide a urine sample	Yes		
**Novel/ technological support**		Yes	
**Waived**			
**Medication offered**			
**MOUD: Buprenorphine s/l**	Yes	Yes	Yes
**MOUD: Methadone**	Yes		
**MOUD: Buprenorphine injectable depot**			

**MOUD: only following in-person visit**			
**Harm reduction interventions**			
**Harm reduction interventions: Naloxone**			
**Harm reduction interventions: Overdose awareness and response**			
**Poly-substance use (co-occurring SUD such as amphetamine and cocaine) or alcohol**			
**Harm reduction interventions: sterile injection equipment**			
**Harm reduction interventions: BBV testing**			
**Harm reduction interventions: HCV cure**			
**Harm reduction interventions: ART- HIV suppression/ PrEP**			
**Harm reduction interventions: Woundcare**			
**Harm reduction interventions: fentanyl strips**			
**Harm reduction interventions: Digital overdose response system**	Yes		
**Not described**		Yes	Yes
**Linking to community MOUD services**	Yes		
**Meeting non-clinical needs**			
**Meeting non-clinical needs: social worker**		Yes	
**Meeting non-clinical needs: Psychosocial assessment**		Yes	
**Engagement**			
**Peer support**		Yes	
**Shared decision making**			
**Round the clock emergency support**	Yes		Yes
**Non-MOUD therapy**			
**Psychosocial/behavioural therapy**		Yes	
**Virtual group or individual therapy**	Yes	Yes	
**Mutual aid**	Yes		

**Diagnosis and treatment of behavioral, psychological, and psychosocial disorders** including depression, anxiety and insomnia.			Yes

Supplementary Table 5: Service characteristics (remaining)

Service characteristics	The Talbot County Detention Center, Maryland.	Respectiul and Equitable Access to Comprehensive Healthcare (REACH Program, Mount Sinai Hospital, New York	Rural MOUD Telemedicine in Primary Care Phase 1 (Feasibility): NCT04418453	The Recovery, Empowerment, Social Services, Prenatal care, Education, Community and	Telemedicine in Addictions service, East Riding Partnership Community Drug and	The Temple Recovery Using Scientific Treatments (TRUST) Clinlc, Philadelphia	Wells House Intensive Outpatient Treatment Program. Marvland	Emergency department callback pilot proiect. Rhode Island	New York City Health + Hospitals H* H opioid Bridge clinic	NULease Medical Solutions, Louisville, Kentucky	Keystone Healh. Pennsvivania	Treatment of Opioid Use Disorder in Pregnant Women via Telemedicine, Charleston, Medical	Comprehensive Opioid Addiction Treatment (COAT) Program, West Virgina	OneFitteen / Samaritan Behavioral Health, Inc. Dayton, Ohio.
**References**	Belcher et al., 2021	Dunham et al., 2021	Hser et al., 2021	Patton et al., 2021	Mayet et al., 2021; 2023	O'Gurek, 2020	Weintraub et al., 2018; 2021	Wightman et al., 2021	Tofighi et al., 2022a, 2022b. Flavin et al., 2022	Cales et al., 2022	Kaur et al., 2022	Guille et al;., 2021	Zheng et al., 2017	Gainer et al., 2023
**Geographic setting**														
**Urban**		Yes		Yes		Yes		Yes	Yes					Yes
**Rural**	Yes		Yes				Yes				Yes		Yes	
**Mixed**					Yes					Yes		Yes		
**Threshold of care**														
**Threshold of care: High**							Yes			Yes			Yes	
**Programmatic rules prioritized**													Yes	
**Abstinence as primary treatment goal**										Yes			Yes	
**Buprenorphine discontinuation for use of other substances**													Yes	
**Medication pick-up or visit frequency based on rigid protocol**													Yes	
**Intensive counseling required to receive buprenorphine**													Yes	
**Mutual aid meetings required to receive buprenorphine**													Yes	

**No availability in non-traditional settings Eligible patients referred to medical office or opioid treatment program**										Yes				
**Threshold of care: Low**									Yes			Yes		Yes
**Same-day treatment entry Prescription at first visit**								Yes	Yes			Yes		
**Home-induction of available**								Yes	Yes			Yes		
**Patient's goals prioritised: Non-judgmental attitude**		Yes						Yes				Yes		
**Patient's goals prioritised:Reduction in illicit opioid use as acceptable goal**								Yes				Yes		
**Use of other substances does not result in treatment cessation**														Yes
**Flexible- Rapid re-initiation of treatment if missed visit**									Yes					
**Availability in non-traditional settings**								Yes				Yes		Yes
**Threshold of care: Unclear**	Yes			Yes	Yes		Yes							
**Enrolment criteria/ philosophy**														
**Inclusion health focus:**									Yes			Yes		Yes
**Service setting**														
**Service setting: Other community settings**					Yes						Yes			
**Service setting: Emergency departments**								Yes						Yes
**Service setting: Hospital based (including obstetrics)**												Yes		
**Service setting: Primary care**		Yes	Yes			Yes			Yes		Yes			

**Service setting: Dedicated treatment facility**										Yes	Yes		Yes	Yes
**Service setting: Prisons**	Yes													
**Type(s) of care**														
**Medical case management:**				Yes	Yes				Yes			Yes		Yes
**Bridge clinic model:**								Yes	Yes					Yes
**Crisis stabilisation** (acutely intoxicated/ in withdrawals)														Yes
**Coordinated Care:**		Yes	Yes	Yes					Yes	Yes		Yes		Yes
**Partnership working (also integrated care):**				Yes		Yes			Yes			Yes		Yes
**Multi-disciplinary Care:**		Yes		Yes								Yes		
**Chronic Care:**			Yes											Yes
**Physician centric or service efficiency focussed**					Yes									
**Office-based opioid treatment (OBOT):**		Yes	Yes	Yes		Yes			Yes	Yes	Yes			Yes
**Opioid Treatment Programs: non-residential**													Yes	
**Opioid Treatment Programs: Intensive outpatient**										Yes				Yes
**Opioid Treatment Programs: partial hospitalisation programmes**														Yes
**Telemedicine specific**														
**Telemedicine facilitator**					Yes	Yes			Yes	Yes		Yes		
**Hybrid: All aspects**		Yes	Yes	Yes	Yes	Yes			Yes	Yes		Yes	Yes	Yes
**Audio-only**								Yes						
**Audio and video by choice**											Yes	Yes		Yes
**Video only**													Yes	

**Must have a rationale for telemedicine appointment**										Yes		Yes		
**Patient may choose telemedicine or in person**					Yes									Yes
**Allows new patients (treatment induction)**			Yes			Yes			Yes		Yes	Yes		Yes
**Allows only patients stabilised on MOUD**														
**Allows only patients already with the service**					Yes					Yes			Yes	
**E-consultation to other hospital specialties or primary care**												Yes		
**Dedicated smartphone app**														Yes
**Chat channels to connect with support staff**														Yes
**After hours troubleshooting line**												Yes		
**Toxicology screening process**														
**Clinically indicated only**				Yes	Yes							Yes		
**Contingency management**										Yes	Yes			
**Mandatory random testing**										Yes			Yes	
**Asynchronous**														Yes
**Novel/ technological support**											Yes			
**Waived**											Yes			
**Medication offered**														
**MOUD: Buprenorphine s/l**				Yes	Yes			Yes	Yes	Yes	Yes	Yes	Yes	Yes
**MOUD: Methadone**				Yes	Yes									
**MOUD: Buprenorphine injectable depot**														
**MOUD: only following in-person visit**														

**Harm reduction interventions**														
**Harm reduction interventions: Naloxone**		Yes						Yes	Yes		Yes			Yes
**Harm reduction interventions: Overdose awareness and response**		Yes						Yes	Yes		Yes			Yes
**Poly-substance use (co-occurring SUD such as amphetamine and cocaine) or alcohol**		Yes								Yes				
**Harm reduction interventions: sterile injection equipment**								Yes						
**Harm reduction interventions: fentanyl strips**								Yes						Yes
**Not described**					Yes					Yes		Yes	Yes	
**Linking to community MOUD services**	Yes			Yes				Yes	Yes					
**Meeting other clinical needs**														
**Meeting other clinical needs: Reproductive healthcare and birth control**												Yes		
**Primary care needs (including managing co-morbidities)**						Yes			Yes					
**Mental health needs**				Yes								Yes		
**Chronic pain needs**										Yes				
**Perinatal care**				Yes								Yes		
**Meeting non-clinical needs**														
**Meeting non-clinical needs: social worker**		Yes		Yes					Yes	Yes		Yes		
**Meeting non-clinical needs: Psychosocial assessment**		Yes												

**Meeting non-clinical needs: Housing services**		Yes		Yes										
**Meeting non-clinical needs: Transport**										Yes				
**Engagement**														
**Outreach**					Yes			Yes						
**Patient navigators:**		Yes							Yes					
**Drop in service**														
**Peer support**		Yes						Yes						
**Shared decision making**				Yes										Yes
**Round the clock emergency support**												Yes		
**Non-MOUD therapy**														
**Psychosocial/behavioural therapy**		Yes								Yes	Yes			
**Virtual group or individual therapy**										Yes				
**Mutual aid**										Yes				
**Counselling**										Yes				
